# Microsporidia and invertebrate hosts: genome-informed taxonomy surrounding a new lineage of crayfish-infecting *Nosema* spp. (Nosematida)

**DOI:** 10.1007/s13225-024-00543-w

**Published:** 2024-11-11

**Authors:** Cheyenne E. Stratton, Sara A. Bolds, Lindsey S. Reisinger, Donald C. Behringer, Amjad Khalaf, Jamie Bojko

**Affiliations:** 1 Fisheries and Aquatic Sciences, University of Florida, Gainesville, Florida, 32653, USA; 2 School of Natural Resources, University of Florida, Gainesville, Florida, 32611, USA; 3 Emerging Pathogens Institute, University of Florida, Gainesville, Florida, 32611, USA; 4 Tree of Life, Wellcome Sanger Institute, Cambridge, CB10 1SA, UK; 5 School of Health and Life Sciences, Teesside University, Middlesbrough, TS1 3BA, UK; 6 National Horizons Centre, Teesside University, Darlington, DL1 1HG, UK

**Keywords:** Microsporidia, Parasite, Disease Ecology, Aquatic, Biological-Invasions.

## Abstract

The Microsporidia, an often overlooked fungal lineage, exhibit increasing diversity and taxonomic understanding with the use of genomic techniques. They are obligate parasites infecting a diversity of hosts, including crustaceans. Crustacea are, in essence, ancient insects and their relationship with the Microsporidia is both diverse and convoluted. Relationships between crayfish and their microsporidian parasites display geospatial and taxonomic diversity. Through classical (histological, ultrastructural, developmental) and genomic (phylogenetic, phylogenomic) approaches, we expand the known diversity of crayfish-infecting microsporidia into the genus *Nosema* by describing three novel species from North America: *Nosema astafloridana* n. sp. infecting *Procambarus pictus* and *Procambarus spiculifer, Nosema rusticus* n. sp. infecting *Faxonius rusticus*, and *Nosema wisconsinii* n. sp. infecting *Faxonius propinquus* and *Faxonius virilis*. Additionally, we provide SSU sequence data for further *Nosema* diversity from *Procambarus clarkii* and *Pacifasticus gambelii*. The taxonomy of aquatic crustacean-infecting *Nosema* have been under scrutiny among microsporidiologists - using genomic data we solidify this systematic relationship. Our genomic data reveal phylogenomic divergence between terrestrial insect-infecting *Nosema* and aquatic crustacean-infecting *Nosema* but place our novel species within the *Nosema*. Comparative genomic analysis reveal that *Nosema rusticus* n. sp. is a tetraploid organism, making this the first known polyploid from the genus *Nosema*. Annotation of the genomic data highlight that crayfish-infecting *Nosema* have distinct proteomic differences when compared to amphipod and insect-infecting microsporidians. Alongside the new diversity uncovered and genome-supported systematics, we consider the role of these new ‘invasive’ parasites in biological invasion systems, exploring their relationship with their invasive hosts.

## 1 Introduction

Microsporidia are often overlooked fungal parasites with obligate, intracellular tendencies, which have been found to parasitize a diverse range of hosts, including: mammals, birds, reptiles, protozoans, fish, and arthropods (Murareanu et al. 2021; Bojko and Stentiford 2022; Bojko et al. 2022). Microsporidia can display high host specificity (Willis and Reinke 2022) or be generalists capable of infecting an array of hosts (Stentiford et al. 2019). Crustaceans have been found to host a wide diversity of microsporidian taxa (Bojko and Stentiford 2022). Crayfish (Astacoidea) host eleven species from five formalized genera: *Alternosema, Astathelohania* (= *Thelohania*), *Cambaraspora, Nosema* (= *Vairimorpha*), and *Ovipleistophora* (Moodie et al. 2003a; Moodie et al. 2003b; Moodie et al. 2003c; Pretto et al. 2018; Bojko et al. 2020a; Bojko et al. 2020b; Tokarev et al. 2020; Stratton et al. 2022a; Stratton et al. 2022b; Stratton et al. 2023a; Stratton et al. 2023b). Species from each genus have been found to infect North American crayfish, except for members from the *Nosema*. Yet, *Nosema* species (previously considered *Vairimorpha*) are known to be important parasites of European and Australian crayfish, and parasitize insect, amphipod, and other host groups (Moodie et al. 2003a; Pretto et al. 2018; Bojko and Stentiford 2022).

The *Nosema* are a genus of arthropod-infecting microsporidians with a complex taxonomic history (Tokarev et al. 2020; Bojko et al. 2022). Microsporidians were traditionally classified based on their morphology and life cycle (Sprague 1977); however, with the arrival of molecular and genomic tools, it has become evident that original morphological-based systematics require supporting genetic and ecological data to accurately describe unique species and their evolutionary relationships (Bojko et al. 2022). The *Nosema* recently underwent a taxonomic revision, primarily based on available molecular data which consisted of ribosomal and protein coding sequences (Tokarev et al. 2020). Prior to this, many *Nosema* were classified as members of the *Vairimorpha*. Crayfish-infecting *Nosema* spp. were originally placed in the genus *Vairimorpha* due to their octosporoblastic sporogeny (Moodie et al. 2003a; Pretto et al. 2018; Tokarev et al. 2020). When prioritizing molecular data, these microsporidians clade with the *Nosema*, suggesting that no known crustacean-infecting microsporidia currently belong to the closely related genus *Vairimorpha* (Tokarev et al. 2020).

The *Nosema* represent the oldest group of Microsporidia, with the first formally described microsporidium being *Nosema bombycis* (Nageli 1857). This genus is globally distributed and has been identified in terrestrial and freshwater environments (Tokarev et al. 2020; Bojko et al. 2022; Bacela-Spychalska et al. 2023). Given that *Nosema* infect a diverse range of hosts, exhibit a broad geographic distribution, and are found in various environments, it is likely that each species possesses unique functions to optimize their infection within their host species (Murareanu et al. 2021). This latter characteristic lends some *Nosema* species well to use as biological control agents to manage insect pests (Hill and Gary 1979; Kohler and Hoiland 2001). Considering these factors and recent taxonomic changes among *Vairimorpha* and *Nosema*, it is pertinent to apply genomic and functional (proteomic/metabolomic) analyses to support ongoing taxonomic understanding.

We describe three novel microsporidians – *Nosema astafloridana* n. sp.; *Nosema rusticus* n. sp.; and *Nosema wisconsinii* n. sp. – identified from crayfish across the USA using histological, ultrastructural, phylogenetic, and phylogenomic data. We also correct and expand upon *Nosema* taxonomy within crayfish by using genomic detail and a comparison of the annotated genes/proteins. These novel isolates represent the first cases of crayfish-infecting *Nosema* within North America and all three are involved in the dynamics of a crayfish invasion.

## 2 Material and Methods

### 2.1 Crayfish locality and collection

**Fig. 1:**
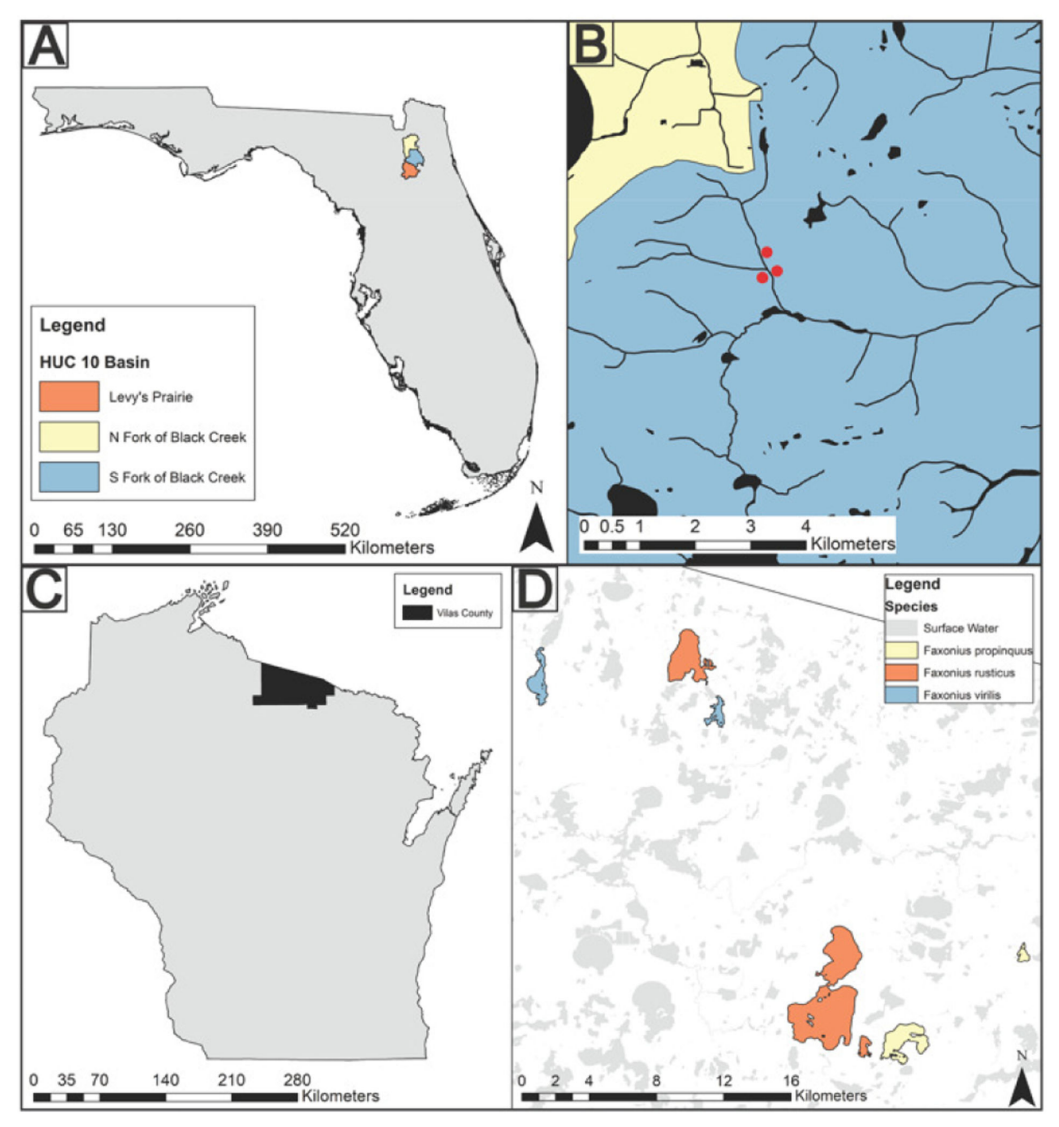
Map of crayfish collection locations. (a) Map of Florida highlighting the three drainage basins (Hydrologic Unit Code 10) in which *Procambarus pictus* were collected. Exact locations are unavailable due to their conservation status. (b) Three sites (red points) in which *Procambarus spiculifer* were collected within the South Fork of Black Creek HUC 10. (c) Map of Wisconsin with Vilas County in black. All Wisconsin hosts were collected within Vilas County. (d) The six lakes Wisconsin hosts were collected from color coded by species of crayfish collected within the lake.

Throughout the midwestern and southeastern USA, 24 crayfish representing seven species were collected for various projects and found to be displaying gross pathology (Table 1, Fig. 1). Signs of a microsporidian infection are visible when the abdominal muscle tissue can be seen through the ventral cuticle, with infected muscle fibers appearing opaque. Most of the crayfish were dissected for histopathology. Eight individuals just had a piece of abdominal muscle tissue fixed in 96% molecular grade ethanol (Table 1).

### 2.2 Histopathology

Crayfish dissected for histopathological screening had biopsies of gut, uropod, muscle, nerve, gonad, heart, hepatopancreas, gill, eye, and antennal gland tissue fixed in Davidson’s Freshwater Fixative (115 mL glacial acetic acid, 220 mL formaldehyde, 309 mL 95% ethanol, 357 mL tap water). After 24–48 hours the fixed tissues were transferred to 70% ethanol. Samples were processed by HistoTech Services (Gainesville, FL) where they were dehydrated, paraffin wax-embedded, sectioned into 3-4μm sections, and stained with hematoxylin and alcoholic eosin. Histology slides were screened with a Leica DM500 microscope (Leica Microsystems, Wetzlar, Germany) and images were acquired using an Olympus BX53 microscope with an integrated Olympus DP74 camera (Evident Corporation, Tokyo, Japan).

### 2.3 Transmission electron microscopy

Abdominal muscle biopsies were obtained from five hosts during dissections and preserved in 2.5% glutaraldehyde in a 0.1% sodium cacodylate buffer for transmission electron microscopy (TEM; Table 1). Sample processing was aided by the use of a Pelco BioWave Pro laboratory microwave (Ted Pella, Redding, CA, USA). Samples were washed in 0.1M sodium cacodylate (pH 7.24) after being transferred to 4% paraformaldehyde with 2.5% glutaraldehyde in 0.1M sodium cacodylate (pH 7.24). The samples were then fixed in 2% osmium tetroxide followed by two water washes. Dehydration of samples was achieved through a graded ethanol series (25% to 100% in 5-10% increments) and later 100% acetone. A ARALDITE/Embed epoxy resin and Z6040 embedding primer (Electron Microscopy Services [EMS], Hatfield, PA, USA) was used to resin infiltrate samples in increments of 3:1, 1:1, 1:3 anhydrous acetone:ARALDITE/Embed and finally 100% ARALDITE/Embed. Semi-thin sections (500nm) were stained with toluidine blue after curing at 60°C for 72 hours. These sections were then cut ultra-thin and stained with 2% aqueous uranyl acetate and lead citrate (EMS, Hatfield, PA, USA) after collection on carbon-coated Formvar 100 mesh grid (EMS, Hatfield, PA, USA). Stained grids were examined using a FEI Teenai G2 Spirit Twin TEM (FEI Corp., Hillsboro, OR, USA) and digital images were captured using Digital Micrograph software with a Gatan UltraScan 2k x 2k camera (Gatan Inc., Pleasanton, CA, USA). ImageJ software was used to obtain morphology measurements from TEM images (Schneider et al. 2012).

### 2.4 Molecular diagnostics

Abdominal muscle biopsies for each individual in Table 1 were preserved in 96% molecular-grade ethanol for molecular diagnostics during dissections. DNA was extracted from muscle biopsies using Qiagen’s DNEasy kit (Qiagen, Hilden, Germany) following the manufacturer’s protocol. DNA extracts were used in 50 μL reaction PCR (Promega ‘Flexi-Tag’) consisting of 1 μM forward primer V1F (5’-CACCAGGTTGATTCTGCCTGAC-3’), and 1 μM reverse primer MC3r (5’-GATAACGACGGGCGGTGTGTACAA-3’), 10 μL flexi-buffer, 0.25 μL Promega Taq polymerase, 1 mM dNTPs, and 2.5 mM MgCl_2_ (Ovcharenko et al. 2010). The reactions underwent an initial denature for five minutes at 94°C then 35 cycles of 94°C denaturation for one minute, 55°C annealing for one minute, and 72°C elongation for one minute followed by a 7-minute final extension period at 72°C.

A primer set was developed for the microsporidium infecting *F. rusticus* and was tested against microsporidians from the following host species: *Faxonius propinquus, Faxonius virilis, Procambarus pictus*, and *Procambarus spiculifer*. The PCR reactions included 1 μM forward primer NrF (5’-CTTGGACCAGACTAATAAACTTCAA-3’) and 1 μM reverse primer NrR (5’-CTTTAGAATCTGCAGATGGTAAAGGC-3’) targeting a hypothetical gene (OR909908-OR909912). The reactions then underwent an initial denaturation at 94°C for five minutes followed by 35 cycles of 94°C denaturation for one minute, 62°C annealing for one minute, and 72°C elongation for one minute followed by a final extension period at 72°C for seven minutes.

Gel electrophoresis was used to visualize amplicons on a 1.5% agarose gel. The resulting amplicons using the V1F and MC3r primers were ~1100 bp while the amplicons using the NrF and NrR primers were ~700 bp. Bands were excised from the gel and extracted using Qiagen’s gel extraction kit. The resulting extractions were sent for Sanger sequencing to Eurofins Genomics (Louisville, KY, USA).

### 2.5 Phylogenetics, haplotyping, and genetic comparisons

A maximum-likelihood (ML) phylogenetic tree was constructed to compare the SSU rRNA gene of our novel isolates (n=25) to all known *Nosema* isolates (n=115) and 3 *Vairimorpha* isolates as an outgroup. The SSU sequences were MAFFT aligned in CIPRES which resulted in 4670 comparative columns including gaps (Miller et al. 2011; Katoh and Standley 2013). Due to the high similarity of the sequences, there were no ambiguous aligned regions, and the gaps within the alignment were minimal. The alignment was analyzed for the best fitting model using the IQ-TREE server which resulted in a tree based on the evolutionary model TIM+F+I+G4, according to Bayesian information criterion (Nguyen et al. 2015; Trifinopoulos et al. 2016). The final tree was constructed using ML process with 1000 bootstrap replicates and annotated in CLC Genomics Workbench v22.0.1. Genetic similarity of all SSU isolates previously mentioned (n=143) were compared using the sequence demarcation tool v.1.2. (Muhire et al. 2014). These same SSU sequences were analyzed using a haplotype network constructed using a minimum spanning network in PopART (Leigh and Bryant 2015).

Additionally, a ML phylogenetic tree was constructed to compare the largest subunit of RNA polymerase II (RPB1) gene of our novel isolates (n=5) to all known *Nosema* isolates (n=12) for which genetic data for the RPB1 gene were available, with *Vairimorpha* (n=2) used as an outgroup (Tokarev et al. 2020; Bacela-Spychalska et al. 2023). The RPB1 sequences were analyzed as described above with an alignment resulting in 5130 comparative columns including gaps. The best fitting model according to Bayesian information criterion was identified as TIM2+F+I+G4 by the IQ-TREE server (Nguyen et al. 2015; Trifinopoulos et al. 2016). The resulting tree was annotated in CLC Genomics Workbench v22.0.1 after being constructed using ML process with 1000 bootstrap replicates. Genetic similarity of these 19 RPB1 sequences were compared using the sequence demarcation tool v.1.2. (Muhire et al. 2014).

### 2.6 Comparative genomics, predicted proteomics, and phylogenomics

DNA extracts from 5 *Nosema-*infected individuals (*F. propinquus, F. virilis, F. rusticus, P. pictus*, and *P. spiculifer*) were submitted to Novogene (Sacramento, CA, USA) for shotgun sequencing. The extracts were prepared into a library using an NEBNext® Ultra™ DNA Library Preparation Kit (PE150) according to manufacturer’s protocol, and sequenced on an Illumina NovaSeq platform (output data: Table 2). The data were trimmed using Trimmomatic (LEADING:3 TRAILING:3 SLIDINGWINDOW:4:15 MINLEN:36) (Bolger et al. 2014) and assembled using SPades v.3.15.3 (phred-offset 33) (Bankevich et al. 2012) separately (output statistics: Table 2). In addition, longer-read sequencing was conducted on the DNA extract from *F. rusticus* to aid with SPades assembly. Sequencing was conducted using a MinION mk1b (Oxford Nanopore). Library preparation was carried out using the Rapid Ligation Sequencing Kit (SQK-RAD004). The library was quantified using the QuBit (broad-range) prior to loading onto a R10.2 flowcell. Basecalling was conducted using MinKNOW v4.1.22.

The contigs from each assembly were subjected to an initial blastx screen, where microsporidian proteins (NCBI, taxid:6029) were used to identify contigs harboring microsporidian genes. This included all predicted proteins from the genomes of: *Nosema bombycis* (GCA000383075.1), *Nosema antheraeae* (SilkPathDB; PRJNA183977), *Nosema granulosis* (GCA015832245.1), *Vairimorpha ceranae* (GCA000988165.1), *Vairimorpha apis* (GCA000447185.1), *Vairimorpha muscidifuracis* (GCA028335825.1) and *Vairimoprha* sp. YNPr (SilkPathDB). The microsporidian contig list for each sample was then annotated using GeneMarkS ‘intronless eukaryotic’ (Besemer et al. 2001) and any host sequences were removed after a blastp check. A secondary blast database was then created from all of the newly annotated microsporidian proteins. This database was used to detect further microsporidian contigs from each sample using a second blastx sweep, identifying any that may have been missed on the first screen. Finally, metaxa2 was used to identify the SSU genes for the microsporidia in each host, adding one additional contig (Bengtsson-Palme et al. 2015). In addition, host sequences and contamination from other sources was detected and removed, as well as corroborating the list of microsporidian contigs, using BlobToolKit (Challis et al. 2020). The microsporidian contigs identified from each sample were then mapped against their corresponding trimmed data in CLC genomics v.12. (Qiagen) to avoid potential chimeric sequences. Genome completeness was assessed using BUSCO v.5.3.0 (Seppey et al. 2019). The microsporidian contigs for each sample can be located at NCBI accessions: JBAOIY000000000 (*F. propinquus* isolate); JBAOIZ000000000 (*F. virilis* isolate); JBAOJA000000000 (*F. rusticus* isolate); JBAOJB000000000 (*P. pictus* isolate); JBAOJC000000000 (*P. spiculifer* isolate). Bioproject numbers: PRJNA1076480 (*F. propinquus* isolate); PRJNA1076481 (*F. virilis* isolate); PRJNA1076482 (*F. rusticus* isolate); PRJNA1076483 (*P. pictus* isolate); PRJNA1076484 (*P. spiculifer* isolate). Biosample numbers: SAMN39942560 (*F. propinquus* isolate); SAMN39942596 (*F. virilis* isolate); SAMN39942614 (*F. rusticus* isolate); SAMN39942679 (*P. pictus* isolate); SAMN39942680 (*P. spiculifer* isolate).

We also estimated ploidy in each of the microsporidian genomes by generating a k-mer spectrum using Jellyfish (v.2.2.10) with k-mers of length 21 (Marçais et al. 2011), and then analyzed the spectra with Genomescope2 (v.2.0) and Smudgeplot (v.0.2.5) (Ranallo-Benavidez et al. 2020). Ploidy estimates were considered reliable only if the monoploid coverage was greater than 20-fold, and we excluded k-mer spectra which showed high levels of contamination or low coverage (Khalaf et al. 2024).

Predicted protein sequences were searched for Pfam domains using InterProScan v.5.60-92.0 (cut-off: 1.0e^-20^) (Jones et al. 2014). These protein domains were listed and quantified to assess their diversity and abundance for each microsporidian, including additional available genomes. R v.4.3.0 (R Core Team, 2013) and RStudio v.2023.06.0 (Allaire 2012) were used to produce comparative plots from the proteomic data, including the use of ggplot2 (Wickham 2011). These protein sequences were also used to infer a new *Nosema-Vairimorpha* genome phylogeny with the addition of further isolates and their annotated proteins (NCBI/SilkPathDB: GCA_000383075, GCA_015832245, GCF_000988165, GCA_000447185; PRJNA183977; PRJNA325422; GCA_028335825) (Li et al. 2017). OrthoFinder was used to identify shared proteins across the *Nosema* spp. and *Vairimorpha* spp., to produce a MAFFT aligned and concatenated alignment file (Emms and Kelly 2019). This file was run through IQ-Tree, which produced a maximum-likelihood tree based on 1000 bootstraps and the evolutionary model Q.yeast+F+I+G4, predicted by Bayesian information criterion (Nguyen et al. 2015) and viewed in FigTree v.1.4.4. Produced an OrthoANI heatmap using OAT v0.93.0 (Lee et al. 2016).

## 3 Results

### 3.1 Geography, gross pathology, histopathology, and electron microscopy

**Fig. 2:**
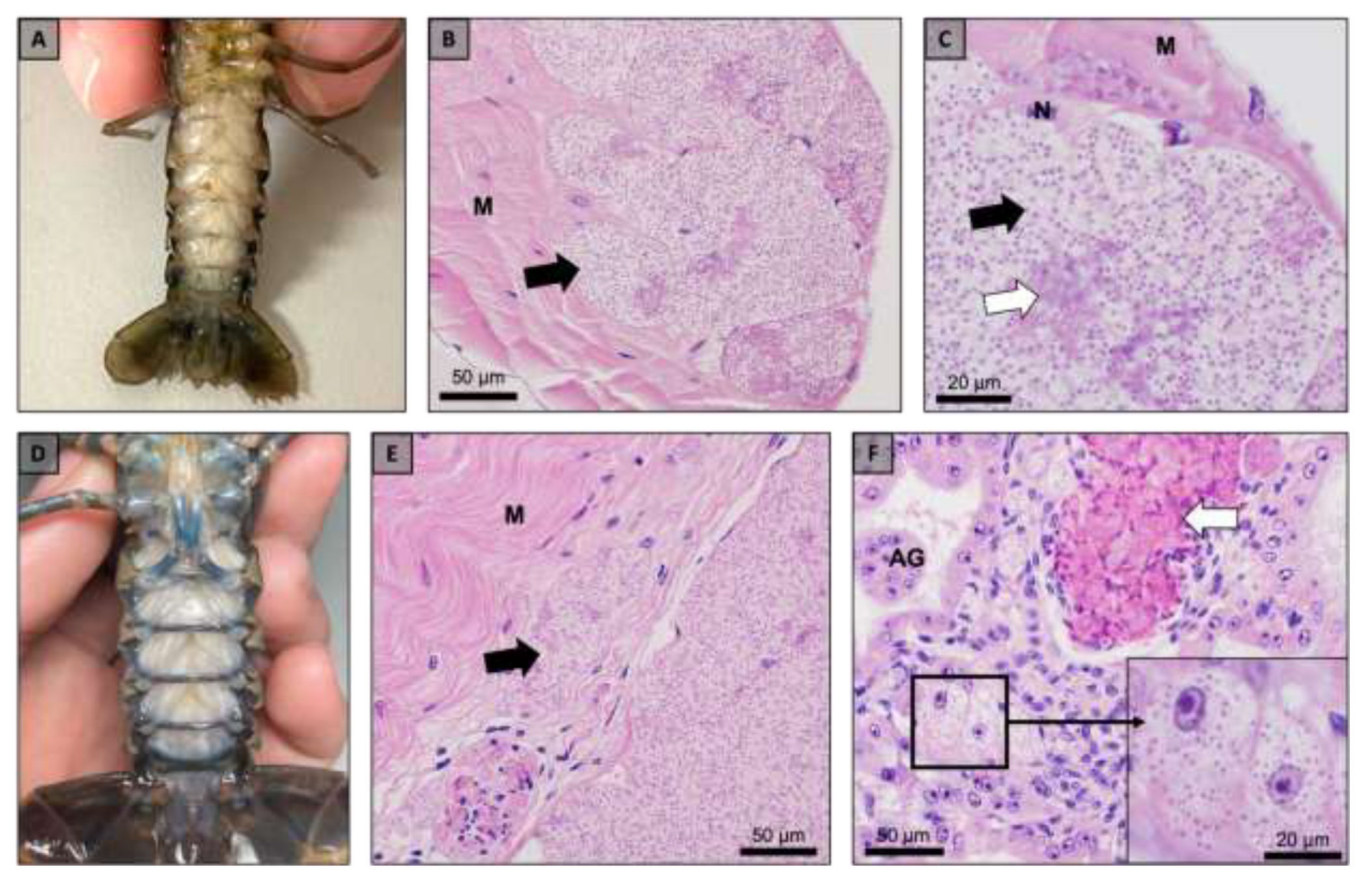
Gross pathology and histopathology of microsporidian infections in Florida crayfish hosts, *Procambarus pictus* (a-c) and *Procambarus spiculifer* (d-f). (a) Muscle tissue of an infected *P. pictus* is opaque and visible through the ventral cuticle of the abdomen. (b) Microsporidian spores (black arrow) develop within muscle tissue (M) of *P. pictus*. (c) Early stage (white arrow) development and mature spores (black arrow) are present within *P. pictus* muscle tissue (M). (d) Muscle tissue of an infected *P. spiculifer* is opaque and visible through the ventral cuticle of the abdomen. (e) Microsporidian spores develop within muscle tissue (M) of *P. spiculifer*. (f) Microsporidian spores were present within the epithelia of the antennal gland (AG) (inset) and an immune response was present resulting in a granuloma (white arrow) in *P. spiculifer*.

Within Florida, seven individuals of the threatened (Florida-listed) species, *P. pictus*, were collected from their native range from the North and South Fork of Black Creek and Levy’s Prairie watersheds and found to be displaying gross pathology (Table 1, Fig. 1a, Fig. 2a). Histopathology revealed microsporidian spores developing within sporophorous vesicles (SPVs) within the sarcolemma of host skeletal muscle fibers (Fig. 2b-c). Additionally, three *P. spiculifer* were collected from their invaded range within the Black Creek drainage (Table 1, Fig. 1b). These individuals displayed gross pathology consistent with a microsporidian infection (Fig. 2d) and histopathology confirmed microsporidian spores were developing within the sarcolemma of host skeletal muscle fibers within SPVs (Fig. 2e). The histopathology of one *P. spiculifer* individual also revealed microsporidian spores developing within the antennal gland (Fig. 2f).

**Fig. 3:**
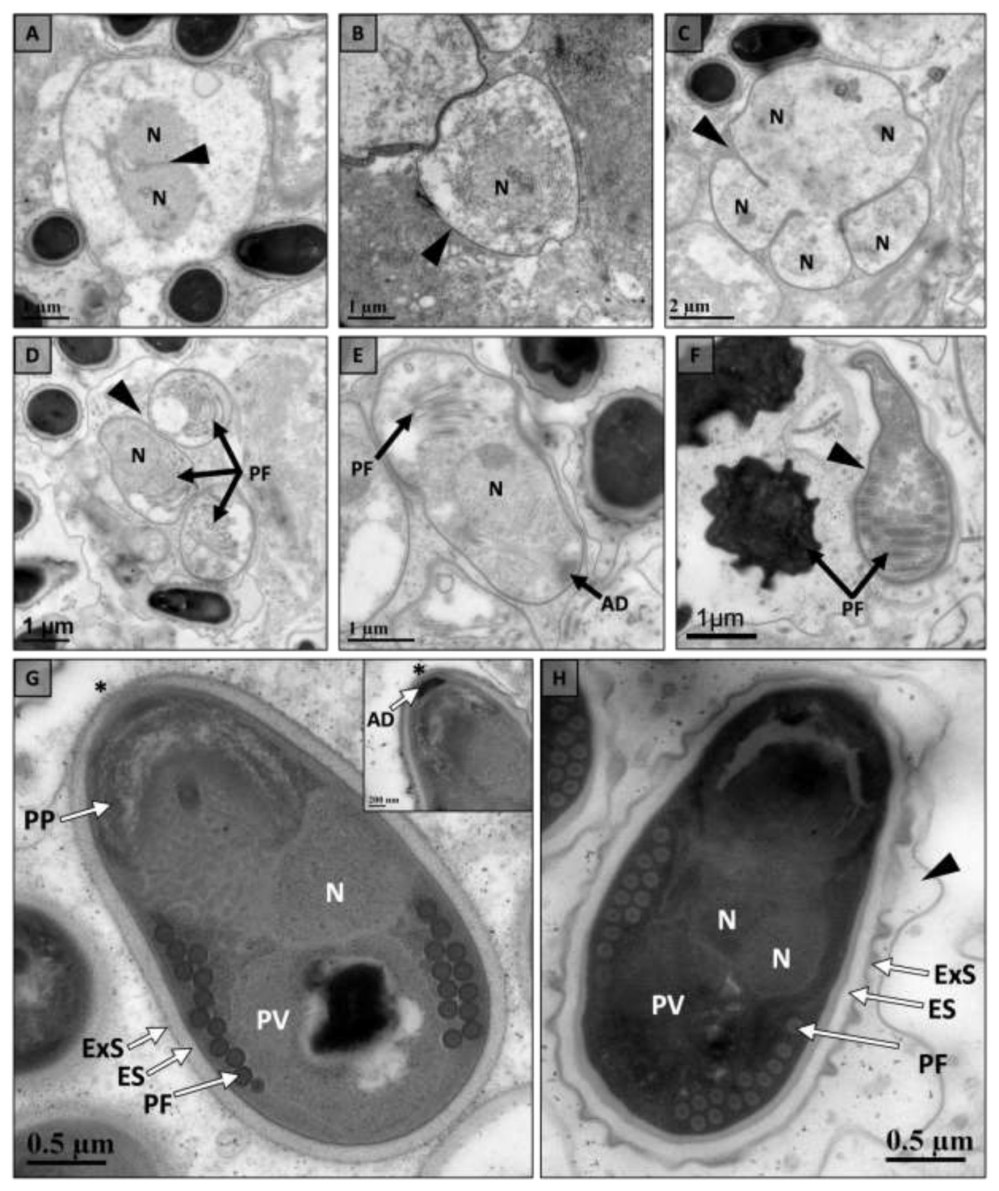
Intracellular developmental cycle of novel microsporidium (*Nosema astafloridana* n. sp.) within the muscle tissue of Florida crayfish hosts (*Procambarus pictus* and *Procambarus spiculifer*). (a) Early development of a diplokaryotic (2N) meront with bound nuclei (arrowhead) in direct contact with host cell cytoplasm. (b) Unikaryotic (N) meront with thickening cell wall (arrowhead). (c) Meront divides into up to eight sporonts through rosette-like division within a sporophorous vesicle (SPV; arrowhead). (d) Unikaryotic sporoblasts within an SPV (arrowhead) begin to develop electron dense organelles including the polar filament (PF). (e) As unikaryotic (N) sporoblasts mature they continue to develop organelles including the polar filament (PF) and anchoring disc (AD). (f) Sporoblasts become more electron dense as they mature, and their cell wall thickens (arrowhead). (g) The ultrastructure of spores includes a bilayered polaroplast (PP), polar filament (PF), posterior vacuole (PV), and well-defined spore wall composed of an electron lucent endospore (ES) and electron dense exospore (ExS). The inset shows the anchoring disc (AD) at the apex of the spore and the thinning of the spore wall above the anchoring disc (*). (h) Spores reach maturity within SPVs (arrowhead) with well-defined ultrastructure including an isofilar polar filament (PF), posterior vacuole (PV), and two nuclei (2N). The spore wall is composed of and electron lucent endospore (ES) and electron dense exospore (ExS).

Electron microscopy indicated the microsporidium infecting *P. pictus* and *P. spiculifer* had a similar development cycle and ultrastructure in both hosts (Table 3). Development of the microsporidium was assumed to begin with a unikaryotic meront, but this stage was not observed. The first observed stage was a diplokaryotic meront with bound nuclei developing in direct contact with the host cell cytoplasm (Fig. 3a). The cell wall of the meront then began to thicken to give rise to an SPV (Fig. 3b). The meront underwent rosette-like division within an SPV to form up to eight sporonts (Fig. 3c). Sporonts began to develop organelles including the polar filament (Fig. 3d) and anchoring disc (Fig. 3e). As the sporonts matured through sporogony, they became electron dense and the cell wall thickened to give rise to a well-defined spore wall (Fig. 3f). Mature spores were pyriform in shape and had 9-12 turns of an isofilar polar filament which ended in an anchoring disc at the apex of the spore (Fig. 3g). The ultrastructure also included a bi-layered polaroplast, posterior vacuole, diplokaryotic nucleus, spore wall composed of an electron lucent endospore and electron dense exospore which thinned at the apex of the spore above the anchoring disc (Fig. 3g-h). The mature spore of *P. pictus* measured 2.77 ± 0.15 um (n=10, SD) in length and 1.49 ± 0.13 um (n=10, SD) in width with a spore wall composed of an endospore measuring 87 ± 11 nm (n=10, SD) and exospore measuring 32 ± 5 nm (n=10, SD) (Table 3). The spore had an isofilar polar filament with a diameter of 114 ± 12 (n=10, SD) (Table 3). The mature spore of *P. spiculifer* had a similar morphology measuring 2.79 ± 0.39 um (n=9, SD) in length and 1.58 ± 0.25 (n=9, SD) in width with 9-12 turns of an isofilar polar filament with a diameter of 121 ± 7 nm (n=10, SD) (Table 3). The spore wall had an endospore measuring 87 ± 10 nm (n=10, SD) and exospore measuring 24 ± 9 nm (n=10, SD) (Table 3).

**Fig. 4:**
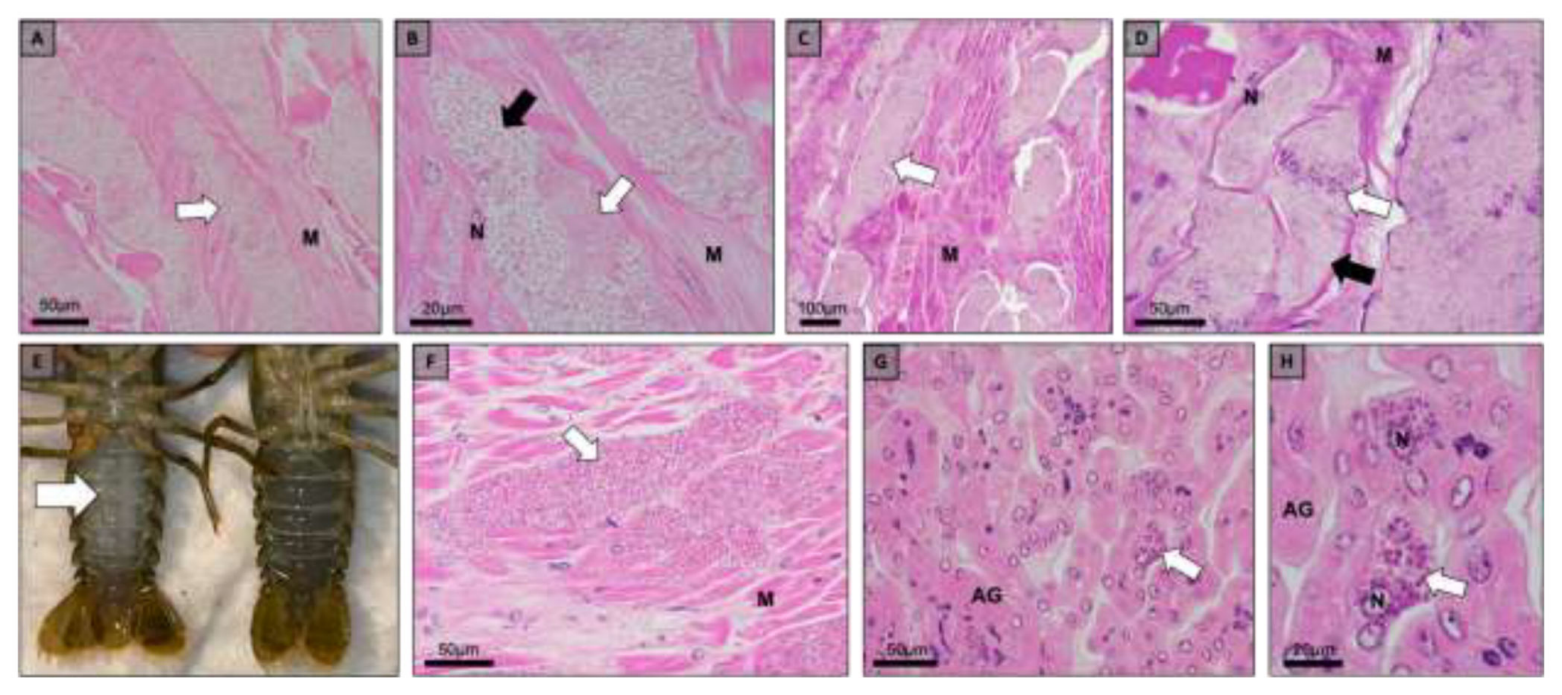
Gross pathology and histopathology of microsporidian infections in Wisconsin hosts, *Faxonius propinquus* (a-b), *Faxonius virilis* (c-d), and *Faxonius rusticus* (e-h). (a) Microsporidian spores (white arrow) develop within muscle tissue (M) of *F. propinquus*. (b) A higher magnification image reveals early development (white arrow) and mature spores (black arrow) developing within the muscle tissue. (c) Muscle tissue (M) of *F. virilis* is infected with clusters of microsporidian spores (white arrow). (d) Early development (white arrow) and mature spores (black arrow) were present within the muscle tissue of *F. virilis*. (e) A microsporidian infected (left) *F. rusticus* with opaque muscle tissue visible through the ventral cuticle (white arrow) of the abdomen compared to an uninfected (right*) F. rusticus* with translucent muscle tissue. (f) Microsporidian spores (white arrow) developing within the muscle tissue (M) of *F. rusticus*. (g) Microsporidian spores (white arrow) developing within the epithelia of the antennal gland (AG) of *F. rusticus*. (h) A higher magnification image reveals hypertrophy of microsporidian infected epithelial cells (white arrow).

Within Northern Wisconsin, crayfish displaying gross pathology consistent with a microsporidian infection were collected. *Faxonius propinquus* (non-native, n=2) were collected from two lakes, *F. virilis* (native, n=2) were collected from two lakes, and *F. rusticus* (invasive, n=8) from three lakes (Table 1, Fig. 1c-d). Histopathology revealed microsporidian spores developing within SPVs within the sarcolemma of the skeletal muscle fibers of each host (Fig. 4a-g). In Trout Lake, WI, a large number of dead individuals were observed near the shore and many crayfish had signs of gross pathology consistent with a microsporidian infection, therefore, we histologically screened 27 *F. rusticus* from Trout Lake and found 48% of the individuals were infected with a microsporidian. A subset of *F. rusticus* from Trout Lake, WI revealed microsporidian spores developing within the antennal gland (Fig. 4g-h).

**Fig. 5:**
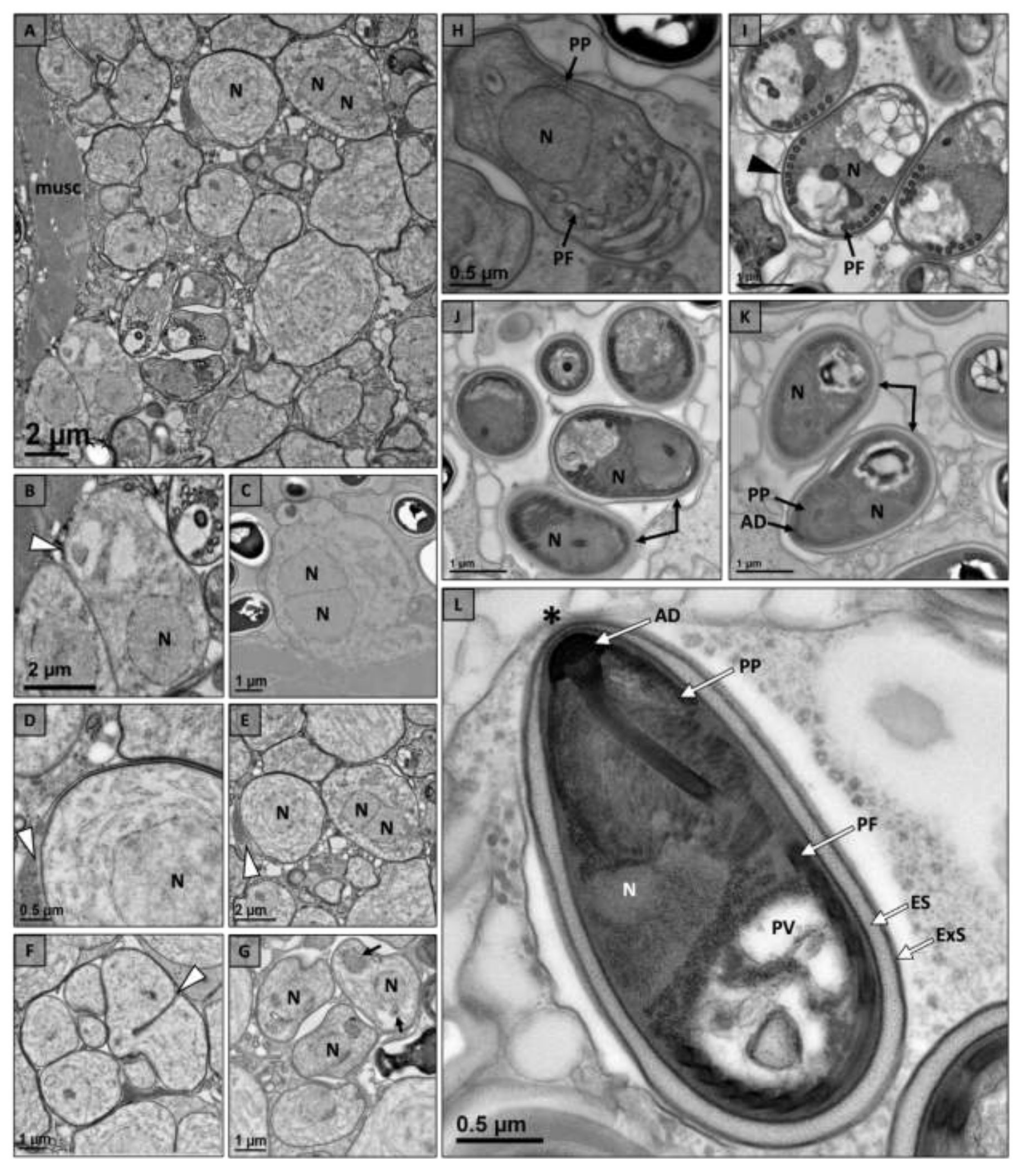
Intracellular developmental cycle of novel microsporidium (*Nosema wisconsinii* n. sp,) within the muscle tissue of Wisconsin crayfish hosts (*Faxonius propinquus* and *Faxonius virilis*). (a) Early development including unikaryotic (N) and diplokaryotic (2N) meronts are found closely associated with host muscle tissue (musc). (b) A unikaryotic meront (N) with a thin cell well (arrowhead) developing in direct contact with host cell cytoplasm. (c) Diplokaryotic meront (2N) with bound nuclei (arrowhead) developing in direct contact with host cell cytoplasm. (d) A high magnification image of a meront developing within a sporophorous vesicle (SPV) (arrowhead). (e) A lower magnification image of the unikaryotic meront (N) developing within a SPV (arrowhead). (f) Meront dividing into eight sporonts within SPV (arrowhead). (g) Early unikaryotic (N) sporonts beginning to develop electron dense organelles (arrows). (h) Early unikaryotic (N) sporoblast beginning to develop the polaroplast (PP) and polar filament (PF). (i) Near mature spores with a thickening electron-dense cell wall (arrowhead) and a well-developed polar filament (PF). (j) As the spores continue to mature the endospore became electron lucent (arrows). (k) Mature spores develop an electron dense layer of a bilayered polaroplast (PP) and anchoring disc (AD). (l) The ultrastructure of a mature unikaryotic (N) spore includes an anchoring disc (AD), a bilayered polaroplast (PP), polar filament (PF) and posterior vacuole (PV). The spore wall has a thick electron lucent endospore (ES) and electron dense exospore (ExS) that is thinnest above the anchoring disc (*).

Based on electron microscopy the microsporidium infecting *F. propinquus* and *F. virilis* had the same morphology and developmental cycle. Development of the microsporidium began with a putative unikaryotic meront in direct contact with the host cytoplasm (Fig 5a-b). The nucleus divided to form a diplokaryotic meront (Fig. 5c) and a sporophorous vesicles (SPV) was formed from the cell wall of the meront (Fig. 5d-e). Within the SPV the meront underwent rosette-like division to form up to eight sporonts (Fig. 5f). Electron dense organelles, including the polar filament and polaroplast, began to form as the sporont progressed through sporogony (Fig. 5g-h). As spores matured a defined spore wall with an electron lucent endospore and electron dense exospore was observed (Fig. 5i-k). The mature spores were unikaryotic with 6-7 turns of an isofilar polar filament (Fig. 5l). Spores were pyriform in shape with a well-defined spore wall composed of an electron lucent endospore and electron dense exospore which thinned at the apex of the spore (Fig. 5l). In *F. propinquus*, mature spores measured 2.79 ± 0.31 um (n=8, SD) in length and 1.57 ± 0.05 um (n=8, SD) in width with 6-7 turns of an isofilar polar filament (diameter 109 ± 4 nm (SD), n=10) (Table 3). Their spore wall was composed of an electron lucent endospore (88 ± 9 nm [n=10, SD]) and electron dense exospore (27 ± 7 nm [n=10, SD]). Similarly, mature spores of *F. virilis* measured 2.75 ± 0.19 um (n=5, SD) in length and 1.49 ± 0.13 um (n=5, SD) in width with 6-7 turns of an isofilar polar filament (diameter 111 ± 5 nm (SD), n=10) (Table 3). Their spore wall was composed of an electron lucent endospore (65 ± 14 nm [n=10, SD]) and electron dense exospore (26 ± 4 nm [n=10, SD]).

**Fig. 6:**
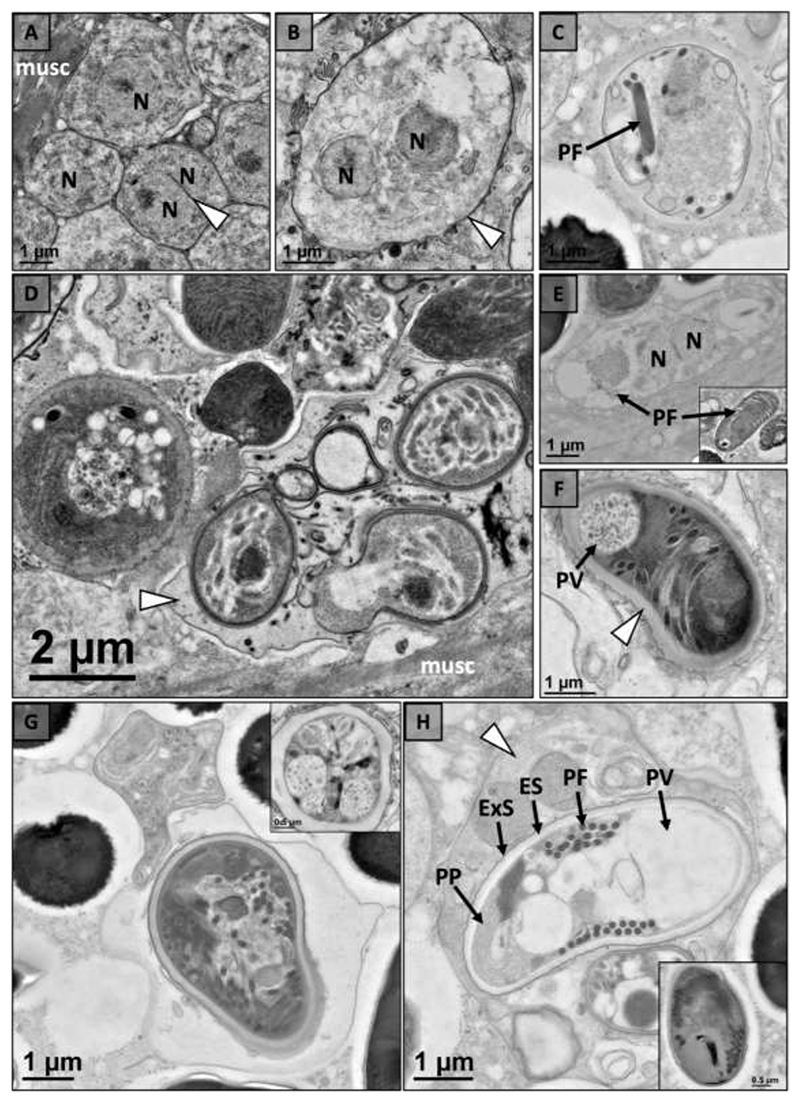
Intracellular developmental cycle of novel microsporidium (*Nosema rusticus* n. sp.) within the muscle tissue of *Faxonius rusticus*. (a) Unikaryotic (N) and diplokaryotic (2N) meronts are found near host muscle tissue (musc). The diplokaryotic meront has bound nuclei (arrowhead). (b) A diplokaryotic meront (2N) with a thickening cell wall (arrowhead). (c) Early sporonts begin to develop a polar filament (PF). (d) Sporoblasts developing within a SPV (arrowhead) in close association with host muscle tissue (musc). (e) Electron lucent diplokaryotic (2N) sporoblast with developing polar filament. Inset shows a sporoblast at a similar stage, but the sporoblast is electron dense. (f) Cell wall of electron dense sporoblast thickens (arrowhead). Sporoblast develops a posterior vacuole (PV). (g) Aberrant spore seemingly developing two polar filaments. Inset shows additional developmental plasticity of the polar filament in a spore with a thick electron lucent endospore. (h) Several spores developing at different rates within a single SPV (arrowhead). The center spore shows details of spore ultrastructure including: polarfilament (PF), bilayered polarplast (PP), and posterior vacuole (PV). The spore wall has a thick electron lucent endospore (ES) and electron dense endospore (ExS). The inset shows another near mature spore that is electron dense.

Electron microscopy of the microsporidium infecting *F. rusticus* revealed similar merogony and early sporogony, but stark differences in the mature spore were found in this host suggesting a different microsporidium infects *F. rusticus* (Table 3). The development of this microsporidium began with a putative unikaryotic meront that underwent nuclear division to form a diplokaryotic meront in direct contact with host cytoplasm (Fig. 6a). The meront cell wall thickened during late merogony (Fig. 6b) and, although not observed, the meront was assumed to undergo rosette-like division to form up to eight sporonts within an SPV as up to five sporonts were observed within an SPV (Fig. 6d). Diplokaryotic sporoblasts with developing polar filaments were observed to be electron-lucent and electron-dense (Fig. 6e). The cell wall of the sporoblast thickened to give way to a defined spore wall as additional structures developed, including the posterior vacuole (Fig. 6f). Developmental plasticity of the microsporidium was observed including a spore that appeared to develop more than one polar filament and a spore with a very thick electron lucent endospore (Fig. 6g). Spores develop to maturity within SPVs with ultrastructure including a bilayered polaroplast, posterior vacuole, diplokaryotic nucleus, and 15-19 turns of an isofilar polar filament (diameter 117 ± 13 nm (SD), n=10) (Fig. 6h). Mature spores were pyriform in shape and measured 4.15 ± 0.39 um (n=3, SD) in length and 1.90 ± 0.19 um (n=4, SD) in width with a spore wall composed of an electron lucent endospore (38 ± 7 nm [n=10, SD]) and electron dense exospore (24 ± 4 nm [n=10, SD]).

### 3.2 Phylogenetics and phylogenomics

The six *P. pictus* and four *P. spiculifer* microsporidian SSU gene isolates were between 33-100% query coverage and 99.70-100% similarity (Online Resource 1; Table 1). The two *F. propinquus* SSU isolates were 98.52% similar (94% query coverage) and *F. propinquus* i1 was most similar to the novel *Nosema* isolate from *F. virilis* i49 (100% query coverage; 99.34% similarity) (Online Resource 1; Table 1). The two *F. virilis* SSU isolates were 98.50% similar (99% query coverage) (Online Resource 1; Table 1). The eight SSU isolates from *F. rusticus* were between 45-100% query coverage and 99.90-100% similarity (Online Resource 1; Table 1). The 1048 bp SSU sequence from *F. rusticus* i26 (OR9338698373) showed 99.81% similarity to the novel *Nosema* isolate from *P. spiculifer* i1 (OR93386991: 100% query coverage; e-value: 0.0). Two additional crayfish species, *Procambarus clarkii* and *Pacifastacus gambelii*, presenting gross pathology consistent with a microsporidian infection were collected; however, tissue was preserved only in ethanol, so these samples are included in the analysis of the SSU gene but absent from other analyses. The 955 bp SSU isolate from *P. clarkii* (OR93386982) showed 99.26% similarity to a *Nosema granulosis* isolate (KM657356: 98% query coverage; e-value: 0.0) from the amphipod host *Gammarus pulex* in Poland. The 853 bp SSU isolate from *P. gambelii* (OR93386983) showed 100% similarity to a *N. austropotamobii* isolate (MF344634: 98% query coverage; e-value: 0.0) from the crayfish, *Austropotamobius pallipes*, in Italy.

**Fig. 7:**
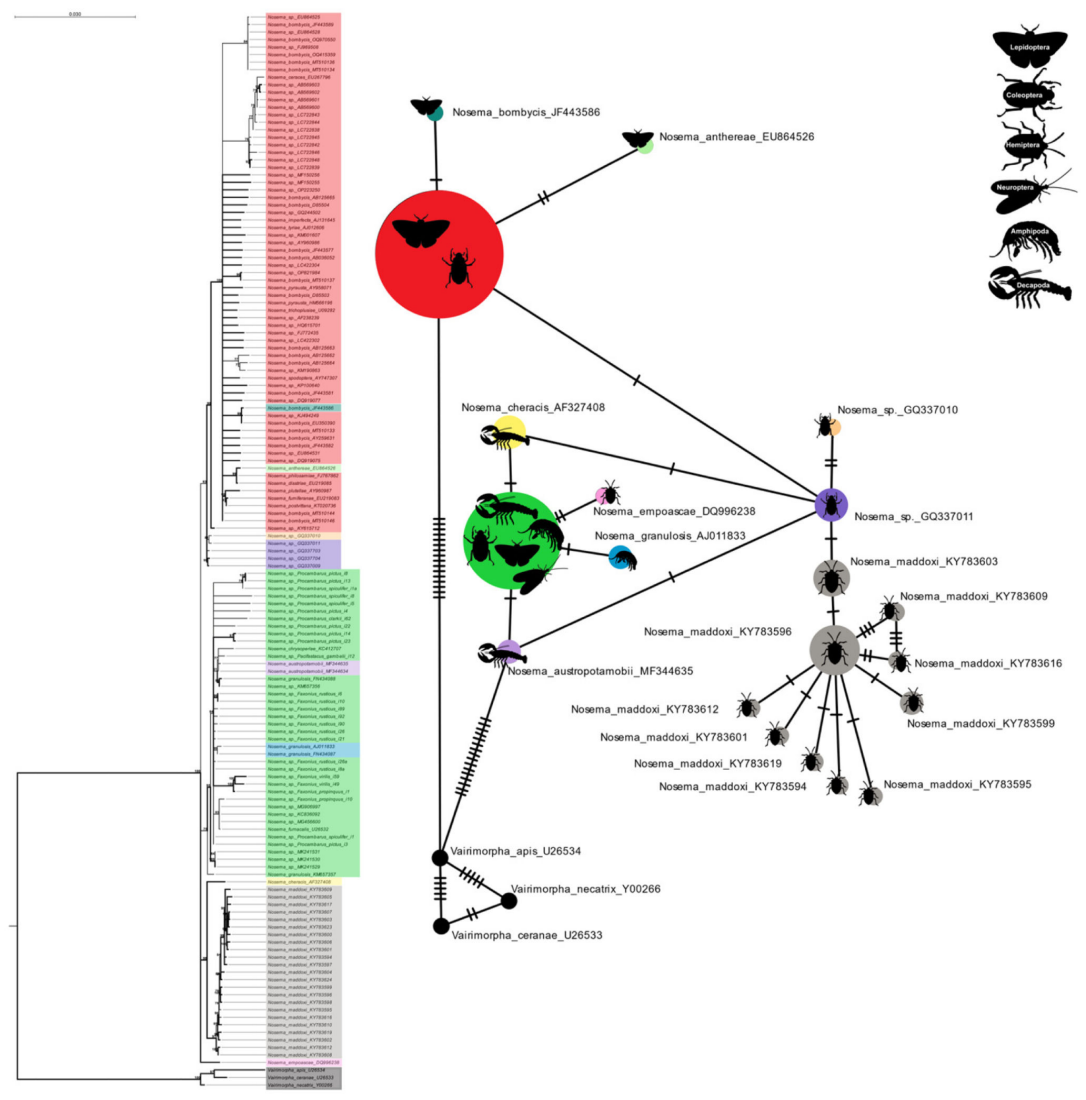
A maximum-likelihood phylogenetic tree of the SSU gene of all *Nosema* isolates (n=140) compared to three *Vairimorpha* isolates. All branches are supported by ≥ 75% bootstrap confidence with thicker branches representing ≥ 90% bootstrap confidence. The haplotype network was produced with the minimum spanning network method in PopART. The size of the circles is proportional to the number of sequences sharing a haplotype. The color and size of each circle of the haplotype network corresponds to the sequences within the phylogenetic tree – larger circles indicate more highly similar isolates occupying the same haplotype. Icons within the circles represent the host groups corresponding to each haplotype. The accession numbers for each sequence are listed in the tree alongside the suggested species name.

The SSU ML phylogenetic tree indicated a clear divergence between the *Nosema* and *Vairimorpha* isolates, with 100% bootstrap support, placing all 25 of our novel microsporidian isolates within the genus *Nosema* (Fig. 7). The SSU tree identified three main groups within the genus *Nosema*: a group of isolates closely related to *N. bombycis*, restricted to terrestrial lepidopteran and coleopteran hosts; a second group with a diverse host assemblage, found in both terrestrial and aquatic environments; and a third group primarily restricted to terrestrial hemipterans (Fig. 7). Because the SSU sequences we analyzed from the crayfish-infecting microsporidia were within 98% similarity to one another, the taxonomic resolution (including bootstrap support) was not great enough to delineate likely taxonomic units (i.e., species boundaries) (Fig. 7). The haplotype network highlights the same issue of high similarity among the *Nosema* isolates, grouping multiple microsporidian species within a single haplotype (Fig. 7). A similarity matrix comparing microsporidian SSU genes further illustrates that the known *Nosema* isolates are all ≥ 96% similar to one another (Online Resource 1). The similarity matrix also highlights the three main groups of isolates within the *Nosema*, represented on the phylogenetic tree (Fig. 7; Online Resource 1).

**Fig. 8:**
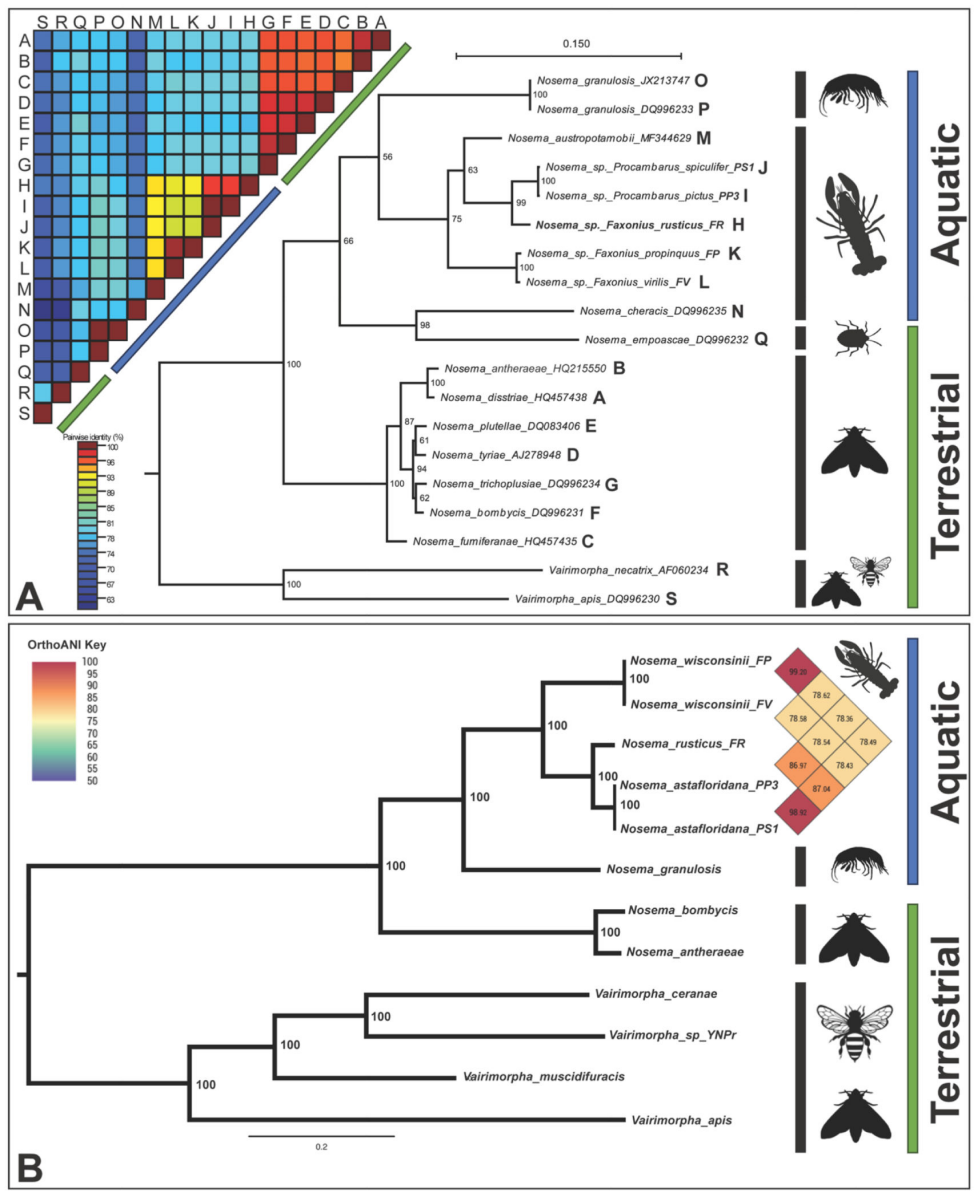
The phylogenetic and phylogenomic placement of our novel crayfish-infecting *Nosema* isolates. (a) A maximum-likelihood phylogenetic tree of the largest subunit of RNA polymerase II (RPB1) gene (nucleotide) of 17 *Nosema* isolates including 5 isolates sequenced in this study. A similarity matrix presents the percent similarity of the RPB1 gene of the isolates presented in the tree, with the letters of the matrix corresponding the letters to the right of each isolate. Host species are abbreviated on the tree (PP3=*Procambarus pictus*; PS1=*Procambarus spiculifer*; FP=*Faxonius propinquus*; FV=*Faxonius virilis*; FR=*Faxonius rusticus*). Accession numbers are included within the tree in the name of each isolate. The host group is indicted by a picture, in order, of a crayfish, an amphipod, a true bug, a moth, or bee. For the matrix and the phylogenetic tree, a colored bar indicates host environment with blue indicating aquatic microsporidia and green indicating terrestrial microsporidians. (b) A concatenated phylogenomic, maximum-likelihood tree, based on 147 single-copy proteins encoded by the genomes of each microsporidian and identified using OrthoFinder. The tree includes proteins from the following genomes: *Nosema granulosis* (GCA_015832245), *Nosema bombycis* (GCA_000383075), *Nosema antheraeae* (SilkPathDB; PRJNA183977), *Vairimorpha ceranae* (GCF_000988165), *Vairimorpha* sp. YNPr (SilkPathDB; PRJNA325422), *Vairimorpha muscidifuracis* (GCA_028335825), and *Vairimorpha apis* (GCA_000447185). Host species are abbreviated on the tree (PP3=*Procambarus pictus*; PS1=*Procambarus spiculifer*; FP=*Faxonius propinquus*; FV=*Faxonius virilis*; FR=*Faxonius rusticus*). Model of substitution: LG+F+I+G4, 371740 amino-acid sites, 1000 bootstraps. Comparative nucleotide similarity across the genomes of crayfish-infecting *Nosema* was conducted using OAT v0.93.1 (Lee et al. 2016). The heatmap represents this comparison and provides the OrthoANI values. The host group is indicted by a picture, in order, of a crayfish, an amphipod, a moth, or bee. Aquatic and terrestrial microsporidians are identified using the right most column.

Due to the high similarity of the SSU gene within the *Nosema* we also developed a ML tree using the RPB1 gene for 19 microsporidians (17 *Nosema*, 2 *Vairimorpha*), since this gene allows for greater microsporidian species delineation (Tokarev et al. 2020; Bacela-Spychalska et al. 2023; Fig. 8a). The RPB1 gene from *P. pictus* and *P. spiculifer* microsporidian isolates were 99.64% similar (100% query coverage) to one another. While the isolates from *F. propinquus* and *F. virilis* were 98.67% similar (100% query coverage) to one another. The *F. rusticus* isolate was 94.01% similar (99% query coverage) to the *P. pictus* and *P. spiculifer* isolates while only 85.06-85.25% similar (99% query coverage) to the *F. virilis* and *F. propinquus* isolates, respectively. The RPB1 ML phylogenetic tree indicates divergence between the *Nosema* and *Vairimorpha* with 100% bootstrap support, and places all our novel isolates within the *Nosema* (Fig. 8a). The analysis identified three distinct branches that were formed by our novel crayfish isolates (100% bootstrap support; Fig. 8a). The RPB1 tree also highlights ‘terrestrial insect-infecting’, and ‘aquatic crustacean-infecting’ groups (aside from *N. empoascae*) within the *Nosema*, with 100% bootstrap support (Fig. 8a). The similarity matrix of the RPB1 gene isolates demonstrates the difference between these two groups, revealing that the terrestrial *Nosema* are within 92% similar to one another, while the aquatic microsporidia are between 70-100% similar to one another (Fig. 8a).

Twelve microsporidian genomes were included in a phylogenomic analysis, including 478 protein comparisons from 8 *Nosema* isolates and 4 *Vairimorpha* isolates (Fig. 8b). The tree distinguished between the *Nosema* and *Vairimorpha* and supported all nodes, at 100 bootstrap support. Our analysis identified three distinct branches formed from the new crayfish isolates, which we have named: *Nosema astafloridana* n. sp. (hosts: *P. spiculifer, P. pictus*), *Nosema wisconsinii* n. sp. (hosts: *F. virilis, F. propinquus*) and *Nosema rusticus* n. sp. (host: *F. rusticus*) [Fig. 8b; Online Resource 2 (taxonomic summary)]. The phylogeny supported the definition of *N. astafloridana* PS and *N. astafloridana* PP as the same species (branch length separation: 0.0012 units; OrthoANI value: 98.92). The phylogeny also supported that *N. wisconsinii* FP and *N. wisconsinii* FV are the same species (branch length separation: 0.0032 units; OrthoANI value: 99.20). Comparatively, *N. rusticus* branched alone (>0.06 units from the other crayfish parasites), but clustered between the two species clusters mentioned above. The phylogeny supported that *N. granulosis* (from *G. roeselii*) was the closest relative outside of the crayfish-infecting species, and that the insect-infecting *Nosema* formed a distinct terrestrial group (Fig. 8b). The *Vairimorpha* branched relatively far away from the *Nosema* (>1.4 cumulative branch length units). All *Vairimorpha* with genomic data, to date, are terrestrial; however, there is distinct phylogenomic separation between the terrestrial *Nosema* and aquatic *Nosema*.

### 3.3 Comparative genomics

**Fig. 9:**
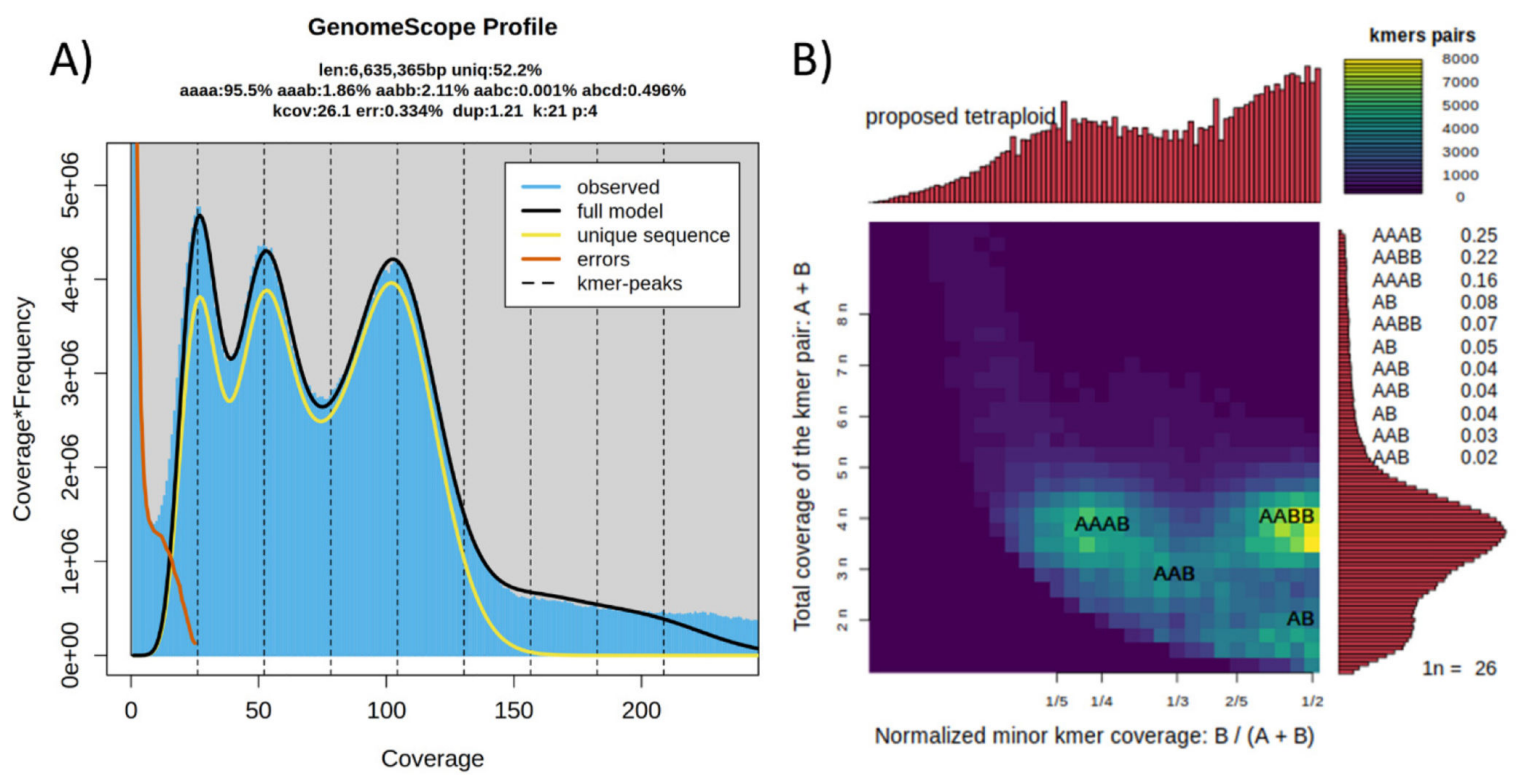
Ploidy estimate for *Nosema rusticus* n. sp. (a) GenomeScope2 plot estimated a haploid genome size of ~6.6 Mbp for *N. rusticus* n. sp., with a tetraploidy model fit. (b) Smudgeplot indicates tetraploidy for *N. rusticus*.

Sequence data analysis for the five *Nosema*-infected individuals using GenomeScope2 and Smudgeplot (Ranallo-Benavidez et al. 2020) revealed that the genome of *N. rusticus* is likely a tetraploid, with an estimated haploid genome size of 6.6 Mbp (Fig. 9), despite a cumulative sequence length of 7.7Mbp (Busco: 57.1%; Table 4). The genome coverage was too low to attain ploidy-level estimates for *N. astafloridana* and *N. wisconsinii* (Online Resource 3). After a BlobToolKit screen (Online Resources 4-5) and decontamination, the haploid assembly sizes ranged between 5.10 and 7.10 Mbp for *N. wisconsinii* and *N. astafloridana* (Table 4). In line with this, BUSCO scores for *N. wisconsinii* and *N. astafloridana* ranged from 93.4 to 94.0% (Table 4).

### 3.4 Comparative proteomics

**Fig. 10:**
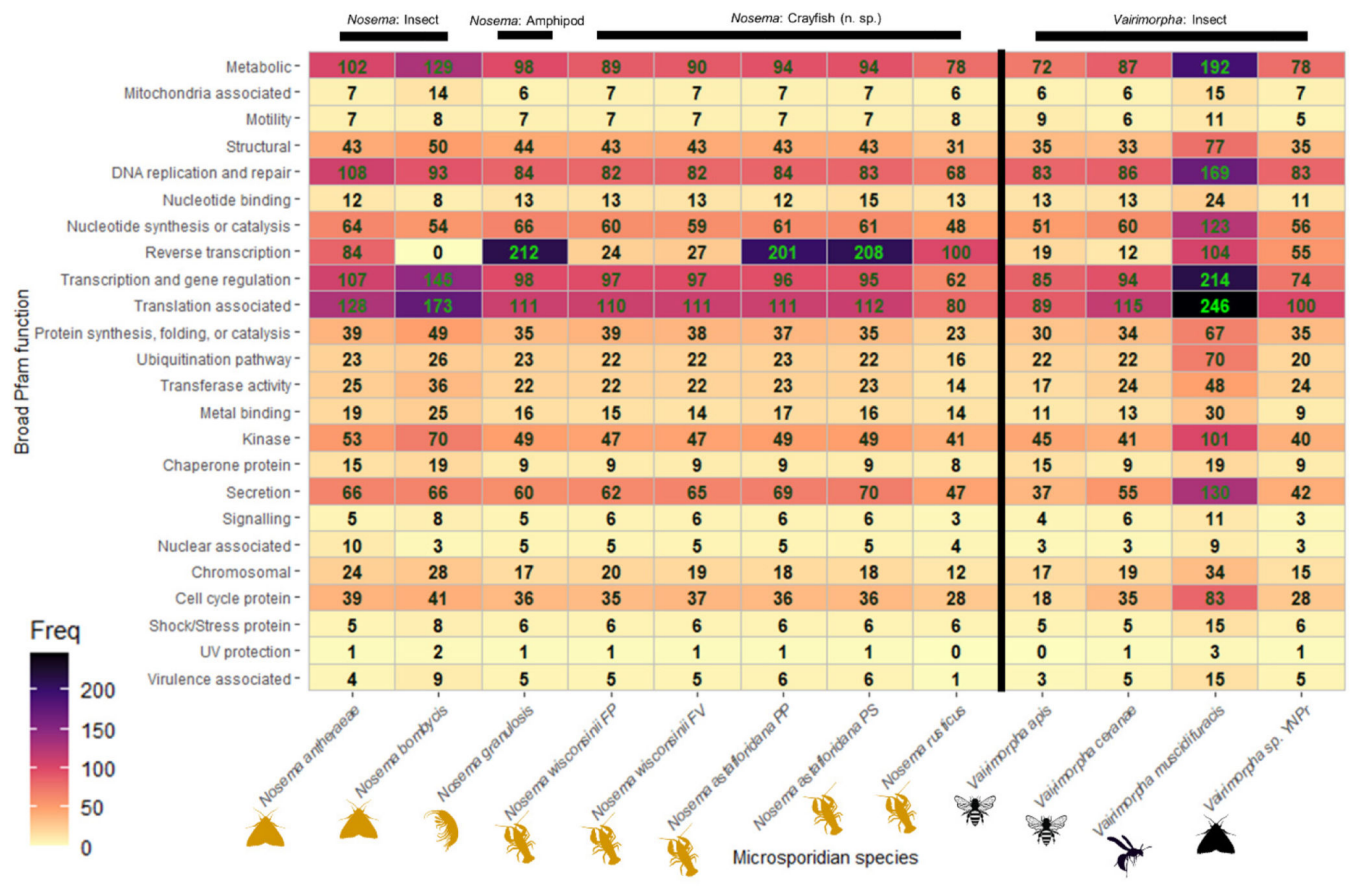
A quantitative heatmap representing Pfam domains encoded within the predicted proteins of each microsporidian genome. Exactly 24 broad functional groups ("Broad Pfam function") were used to develop this overview of the functional proteome of each microsporidium in our assessment. The 'Freq' key indicates how many of these domains were detected in the analysis, and the specific number of domains detected is noted in each box within the heatmap. Small animal icons are provided to indicate the host group – gold icons reflect hosts of Nosema spp., whereas black icons represent hosts infected by *Vairimorpha* spp. A detailed breakdown of each broad functional group is provided in Online Resource 8 (including specific Pfam number(s)), and the data files used to develop the table are located in Online Resources 6 and 7. The original Pfam association was made using the predicted proteome, based on the microsporidian genome sequences explored in this study, via InterProScan v.5.60–92.0 (cut-of: 1.0e-20). The heatmap was developed in Rstudio v.2023.06.0 using the ggplot2 package.

Each protein annotated onto the genomes of the *Nosema* isolates listed in Table 3, all *Nosema* listed in Table 4, as well as *Vairimorpha apis, Vairimorpha ceranae, Vairimorpha* (= *Nosema*) *muscidifuracis*, and *Vairimorpha* sp. VNPr, were scanned using InterProScan to determine the abundance and diversity of Pfam domains contained in their respective predicted proteomes (cut-off: 1.0e^-20^). The analysis resulted in protein domains that clustered into 23 functional groups to aid with understanding their distribution across taxa (mitochondrial, motility, metabolism, cell cycle, shock proteins, signaling, UV protection, structural, chaperone proteins, kinase proteins, chromosomal maintenance, metal binding, DNA replication and repair, nucleotide synthesis and catalysis, nuclear localization, nucleotide binding, protein synthesis, folding, and catalysis, reverse transcription, secretion, transcription, translation, transferase activity, ubiquitination, and virulence), which are compared between species (Fig. 10; Online Resources 6-7).

Overall, the whole range of *Nosema* taxa included in our analysis were all found to encode 7 Pfam domains (PF17030, PF02878, PF13520, PF00265, PF00924, PF01172, PF00572) that appear to be missing from the *Vairimorpha* isolates that we include in our analysis. There were four Pfam domains [PF17004 (microsporidial recognition proteins), PF03124 (signal transduction protein), PF03194 (snRNA/splicing associated) and PF18307 (transcription factor)] specific to crayfish-infecting *Nosema*, only. A further 5 Pfam domains (PF05207, PF01171, PF01650, PF05002, PF17777) were specific only to the *Vairimorpha* spp. in our analysis and were not apparent in the *Nosema*. Overall, there is significant potential to explore this information from a functional and taxonomic standpoint to provide further demarcation among these genera. Detail on these comparisons can be found in Online Resource 8.

## 4 Discussion

The diversity of crayfish-infecting Microsporidia has rapidly expanded in recent years, including species from five genera: *Alternosema, Astathelohania, Cambaraspora, Ovipleistophora*, and *Nosema* (Moodie et al. 2003a; Pretto et al. 2018; Bojko et al. 2020a; Bojko et al. 2020b; Stratton et al. 2022b; Stratton et al. 2023b). We add to this growing diversity by formally describing three new species: *Nosema astafloridana* n. sp.; *Nosema rusticus* n. sp.; and *Nosema wisconsinii* n. sp., using histological, developmental, ultrastructural, genomic, and genetic data (Online Resource 2). In addition to the taxonomic benefit and functional understanding we gain by exploring these pathogens, their hosts (Crayfish) are prominent freshwater invaders, causing extensive economic and ecological damage (Lodge et al. 1994; Twardochleb et al. 2013), and understanding these parasites may provide further insight into their invasion dynamics.

### 4.1 Expanding the *Nosema* using genomic data

The phylogenetic framework for the Microsporidia has recently been updated (including higher taxonomic units: Opisthophagea; and lower taxonomic units), which emphasizes genetic/genomic data supported by morphological, ultrastructural, ecological, and developmental data (Bojko et al. 2022; Galindo et al. 2023). This updated framework replaces the previous clade-based taxonomic system (i.e., I, II, III, IVa, IVb, V) with a classical Linnaean naming system with integrated phylogenetic support, consisting of seven higher taxonomic units within the Microsporidia (Ovavesiculida, Caudosporida, Nosematida, Neopereziida, Glugeida, Enterocytozoonida, and Amblyosporida) (Bojko et al. 2022; Galindo et al. 2023). The Nosematida [previously clade 4a, *sensu* Park and Poulin, (2021)] houses three microsporidian families: Encephalitozoonidae; Heterovesiculidae; and the Nosematidae (Bojko et al. 2022). The Nosematidae house the genera *Nosema* and *Vairimorpha*, which underwent recent redefinition based on phylogenetic data (Tokarev et al. 2020).

Historically, the *Nosema* and *Vairimorpha* were thought to infect only terrestrial insects, namely lepidopterans and honeybees (Tokarev et al. 2020); however, our knowledge of the diversity of *Nosema* within crustaceans is rapidly growing, allowing us to gain a better understanding of its evolutionary relationships with other groups. Based on the phylogenetics of the RPB1 gene tree, and the phylogenomic tree, it is clear that there are two distinct lineages: one currently composed of only terrestrial lepidopteran hosts, and the other primarily infecting aquatic crustacean hosts (ex. terrestrial infecting *N. empoascae*) (Fig. 8). However, this relationship cannot be determined using only SSU data (Fig. 7). The SSU gene does not provide enough taxonomic resolution to be the sole genetic marker used when defining *Nosema* and *Vairimorpha*; therefore, several recent studies have suggested the use of alternative genes such as the RPB1 gene, or the ITS of the rRNA gene, or preferentially, sequencing the entire genome (Tokarev et al. 2020; Bojko et al. 2022). In our study, although fewer genomes are available to represent *Nosema* and *Vairimorpha* diversity than single gene data, we show that multi-protein trees are possibly the most viable method of delineating these taxa phylogenetically. However, our protein domain analysis has outlined a series of unique domains for each of the *Vairimorpha* and *Nosema* taxa that could be followed up on to provide more specific PCR/barcoding targets for taxonomic delineation between the genera.

This study not only elucidated the phylogenetic relationships among *Nosema* microsporidians, but provides new genomic information about this group. The cumulative contig lengths identified for the new crayfish-infecting microsporidian haploid genomes were between 5.41 - 7.77 Mbp; however, we have yet to provide a complete genome for these species, which are missing repeat regions and other hard-to-sequence regions of their genomes. This leaves the contig lengths we provide as lower-end estimates of the total haploid genome size for these individuals. For *N. wisconsinii* and *N. astafloridana*, we were not able to confirm whether these species are polyploids or if their genomes are diploid (Online Resource 2); however, *N. rusticus* was estimated to have a tetraploid genome (Fig. 9a-b). The assembly for *N. rusticus* retrieved a BUSCO score of 57.1%, highlighting the need for a better assembly and further analysis of this polyploid (Table 4). This is the first tetraploid *Nosema* identified to date, adding an additional species to the list of taxa identified as polyploid across the Microsporidia (Pelin et al. 2015; Khalaf et al. 2024).

### 4.2 *Nosema* diversity in crayfish

The diversity of crayfish-infecting *Nosema* has increased as the group’s global presence. The first crayfish-infecting *Nosema, N. cheracis*, was isolated from *Cherax destructor* in Australia (Moodie et al. 2003a). Several years later, *N. austropotamobii* was isolated from *A. pallipes* in Italy (Pretto et al. 2018). We have now established that the *Nosema* include crayfish-infecting microsporidians from the USA as well, with the description of our three novel species. The *Nosema* and *Astathelohania* are the two most diverse genera of crayfish-infecting microsporidia, each with five formally described species (Moodie et al. 2003a; Moodie et al. 2003b; Moodie et al. 2003c; Pretto et al. 2018; Stratton et al. 2022b). Within the *Astathelohania*, a geographic split was observed and the diversification of members of the genus follow the diversification patterns of their crayfish hosts suggesting that the *Astathelohania* co-evolved with their hosts (Stratton et al. 2022b). The *Nosema* do not show the same clear evolutionary pattern and is explored further below.

The RPB1 gene reveals *N. cheracis* from Australia (Family Parastacidae) is most dissimilar from other crayfish-infecting *Nosema*, while North American (Family Cambaridae) crayfish-infecting *Nosema* are as equally dissimilar from each other as they are from *N. austropotamobii* in Europe (Family Astacidae). This suggests *Nosema* infection in crayfish may have evolved in isolation between the Southern (Parastacidae) and Northern (Astacidae and Cambaridae) hemisphere crayfish families (Crandall and De Grave 2017). Crayfish families in the Southern and Northern hemispheres diverged >265 Mya (Bracken-Grissom et al. 2014), yet a clear evolutionary pattern for *Nosema* infecting Northern hemisphere crayfish was not found. *Nosema wisconsinii* is phylogenetically closer to *N. austropotamobii*, which infects Astacidae Family members, than to *N. astafloridana* or *N. rusticus*, both infecting members of the Cambaridae Family (Fig. 8a). Due to the high host specificity observed in some microsporidia we would expect a greater diversity of microsporidians in areas with a higher diversity of host taxa (Weiss and Reinke 2022).

North America is home to the crayfish Cambaridae, the most diverse crayfish family, undergoing radiation and diversification about 90 mya (Reynolds and Souty-Grosset 2012). The greatest diversity of crayfish-infecting *Nosema* exists within North America, infecting members of the family Cambaridae, as we highlight in our study. Obtaining genomic data for *N. cheracis* and *N. austropotamobii* would help determine whether a geographical split or host-parasite co-evolutionary trajectory is more likely to have taken place in the history of the crayfish-infecting *Nosema*. Additional genomic data for more crustacean and insect hosts would aid in understanding the origin and evolution of *Nosema* within arthropods.

Of additional interest is the function of the different *Nosema* parasites across arthropods, and particularly in crayfish. In our results, we outlined a wide range of Pfams distributed across the predicted proteome of the new *Nosema* spp., as well as species that have already had their genomes sequenced (Fig. 10). There were several distinct proteomic differences between the *Nosema* (n=7 unique Pfam domains) and *Vairimorpha* (n = 5 unique Pfam domains). The crayfish-infecting microsporidians and insect-infecting species also show some Pfam domain differences, as well as some differences between the terrestrial hosts and aquatic hosts of microsporidians explored in our results. For example, PF00210 (Ferritin) was only present in the aquatic *Nosema* spp. and missing from all terrestrial *Nosema* (Online Resource 8), suggesting that iron metabolism may not be as necessary in terrestrial systems, and in insect hosts, for these microsporidian parasites. The crayfish-infecting *Nosema* were also found with 4 unique Pfam domains [PF17004 (microsporidial recognition proteins), PF03124 (signal transduction protein), PF03194 (snRNA/splicing associated) and PF18307 (transcription factor)] that were not present in any of the other taxa and may be valuable for delineating these species and better understanding their functional role as crayfish-infecting species.

Several research groups have identified significant differences between the proteomes of different microsporidian parasites from different hosts and environments, from within the same genus, and this information has taxonomic value (Wiredu Boakye et al. 2017; Wadi et al. 2023).

### 4.3 Are these newly described *Nosema* important for crayfish invasions?

Invasive species pose a significant threat to global biodiversity, often exerting profound impacts on recipient communities and ecosystems, leading to substantial economic and ecological damage (Pimentel et al. 2005, Simberloff et al. 2013; Bojko et al. 2023). Crayfish are pervasive freshwater invaders, and because of their ability to reach high densities and ecological role as omnivores, these invasions often result in extensive ecological consequences (Lodge et al. 1994, Twardochleb et al. 2013). Globally, the estimated annual cost of invasive crayfish impacts is $5.7 million (Kouba et al. 2022). However, this figure is recognized as a severe underestimate due to underreported economic data on crayfish invasions (Kouba et al. 2022).

Microsporidian parasites have been implicated in previous crayfish invasions, as have a whole range of invasive parasites (Blakeslee and Moore, 2023; Thieltges and Goedknegt, 2023; Warren et al. 2023; Wood et al. 2023). Invasive signal crayfish (*Pacifastacus leniusculus*) in Britain were infected with the microsporidian *Astathelohania contejeani* at a high prevalence (26–75%) (Dunn et al. 2009). Based on the historical range of *A. contejeani* and sequence similarity between the native and invasive host, it is hypothesized that these parasites were transmitted to invasive *P. leniusculus* from native *A. pallipes* (Dunn et al. 2009). *Nosema austropotamobii* has yet to be isolated from *P. leninusculus*; however, *N. austropotamobii* may be an invasive parasite introduced to Europe by *P. leniusculus* considering we discovered a *Nosema* isolate within *Pacifasticus gambelii* from Wyoming that has a 100% identical SSU sequence to *N. austropotamobii*.

Within this study, each novel microsporidium is involved in an invasion system. *Nosema wisconsinii* infects *F. propinquus* and *F. virilis* within Vilas County, WI. In this region, *F. propinquus* is a non-native species that has replaced *F. virilis* in many lake systems (Lodge et al. 1986; Olsen et al. 1991). Here we may be seeing a parasite spillover event occurring where *F. propinquus* introduced *N. wisconsinii*, which is able to infect *F. virilis* (Power and Mitchell 2004). Alternatively, *F. propinquus* may be acting as a non-native parasite sink, increasing parasite pressure on *F. virilis* through a parasite spillback event (Kelly et al. 2009). Another possibility is a long co-evolutionary history of *N. wisconsinii* with both hosts, given the native ranges of *F. virilis* and *F. propinquus* overlap.

In 2019, we discovered *N. rusticus* within a population of invasive *F. rusticus* in Trout Lake, WI, and the prevalence was relatively high (48%). The discovery included a large number of dead crayfish observed within less than a month, indicating an epizootic was underway. The parasite we analyzed in this study was from a member of this mortality event and has been described as *N. rusticus*. Since *N. rusticus* appears to be the first *Nosema* with a tetraploid genome (Khalaf et al. 2024), we speculate that tetraploidy may impact virulence; however, there are a series of unique genetic and protein markers we identify here that may also play crucial yet unknown roles (e.g. virulence factors). If we explore Khalaf et al. (2024)’s deductions on which microsporidia show tetraploidy, we find several other crustacean-infecting species that drive host mortality. These include the mortality driving *Cucumispora dikerogammari* (form *Dikerogammarus villosus*; Ovcharenko et al. 2010); the mortality driving *Astathelohania contejeani* from *Austropotamobius* sp. (Imhoff et al. 2012); and *Agmasoma penaei*, a pathogen of penaeid shrimp with significant impact on aquaculture (Sokolova et al. 2015). Although a theory at this stage that could be explained by other virulence factors yet undetermined, this may be an avenue for interesting experimentation.

*Nosema rusticus* may regulate the invasive crayfish population, in a similar way to some insect-infecting microsporidia and act as a natural biocontrol agent (Hill and Gary 1979; Kohler and Hoiland 2001). If this microsporidium is able to regulate the population of its invasive host, it may be a candidate for an applied biocontrol agent in future; however, transmission studies need to be conducted to assess whether *N. rusticus* is able to infect native hosts before this can be considered. To date no such hosts have been identified in the wild. Additional studies to assess the ecological impact of this highly prevalent and mortality driving parasite are needed.

Finally, *N. astafloridana* infects an imperiled species, *P. pictus*, that is listed as ‘Threatened’ by the State of Florida, and *P. spiculifer*, which is an invasive species in the Black Creek drainage, FL. The invasion of *P. spiculifer* is thought to be a primary driver of the decline of *P. pictus* (Tripp et al. 2024). This parasite may be relevant to the dynamics of this invasion, since *N. astafloridana* could potentially limit the population size or competitive ability of one or both species, and therefore the presence of the invader might alter the prevalence of microsporidian infections in *P. pictus*. To fully understand the implications of this parasite, determining the original host of the parasite and whether a parasite spillover or spillback event is occurring as a part of this invasion would provide further clarity.

### 4.4 Conclusion

We formally describe the first North American crayfish-infecting members of the genus *Nosema*: *N. astafloridana* n. sp., *N. wisconsinii* n. sp., and *N. rusticus* n. sp., and provide genomic data and accompanying developmental, ultrastructural, pathological, and phylogenetic detail for each novel species. We identified the first polyploid *Nosema* (*N. rusticus*), which happens to be part of an ongoing epizootic, and may indicate that polyploidy, among other factors, could be related to virulence for this microsporidium. We also provide an overview of the proteome between/within the *Nosema* and *Vairimorpha* species, which further highlights differences between the two ‘hot-topic’ genera, providing further potential taxonomic delineation loci and protein targets.

Each novel parasite presented here is associated with a crayfish invasion within the US. In one case, we identify a new *Nosema* isolate from *P. gambelii*, which shows 100% similarity to *N. austropotamobii*, possibly highlighting the origin of this European pathogen as an invader from the USA. The ecological role of these *Nosema* within invasion dynamics, and common traits across these ecosystems that may help us to better understand the broader role of parasites in biological invasions, is needed.

## Supplementary Material

Online Resources

Online Resources 2, 6, 7 and 8 Legend

## Figures and Tables

**Table 1:** Information related to each crayfish’s locality, method of collection, sex, carapace length, and available microsporidian data (SSU= rRNA SSU sequence; Hist = Histology; Genome = Genomic data; TEM = Transmission Electron Microscopy). – indicates data are unavailable. *Procambarus pictus* is a Florida state-listed threatened species, therefore, the exact location of their collection cannot be made available.

Species (Isolate)	Site	Coordinates	Invasion Status	Collection Method	Date of Collection (mm/dd/yyyy)	Sex	Carapace Length (mm)	SSU	Hist	Genome	TEM	Accession Number
*Faxonius propinquus* (i1)	Big Muskellunge Lake, WI	46.00851, -89.62941	Non-native	Hand Collection	06/21/2019	Female	30	✓	✓	✓	✓	OR933869^am^; OR909904^bm^; OR909908^cm^
*Faxonius propinquus* (i10)	White Birch Lake, WI	46.06022, -89.52094	Non-native	Hand Collection	06/22/2019	Male	22	✓	✓	–	–	OR933870^am^
*Faxonius rusticus* (i26)	Trout Lake, WI	46.01913, -89.65534	Invasive	Hand Collection	06/20/2019	Female	25	✓	✓	✓	✓	OR93386973^am^; OR93386973^ag^; OR909903^bm^; OR909909^cm^
*Faxonius rusticus* (i8)	Trout Lake, WI	46.03913, -89.67834	Invasive	Hand Collection	06/20/2019	Male	30	✓	✓	–	–	OR93386975^ag^
*Faxonius rusticus* (i6)	Trout Lake, WI	46.01913, -89.65534	Invasive	Hand Collection	06/20/2021	–	–	✓	–	–	–	OR93386976^am^
*Faxonius rusticus* (i10)	Trout Lake, WI	46.03913, -89.67834	Invasive	Hand Collection	06/20/2019	–	–	✓	–	–	–	OR93386977^ag^
*Faxonius rusticus* (i21)	Presque Isle Lake, WI	46.21882, -89.77151	Invasive	Hand Collection	06/30/2023	Male	26	✓	✓	–	–	OR93386978^am^
*Faxonius rusticus* (i89)	Little John Lake, WI	46.01974, -89.64431	Invasive	Hand Collection	07/24/2023	Male	16	✓	–	–	–	OR93386979^am^
*Faxonius rusticus* (i90)	Little John Lake, WI	46.01974, -89.64431	Invasive	Hand Collection	07/24/2023	Female	23	✓	–	–	–	OR93386980^am^
*Faxonius rusticus* (i92)	Little John Lake, WI	46.01974, -89.64431	Invasive	Hand Collection	07/24/2023	Male	20	✓	–	–	–	OR93386981^am^
*Faxonius virilis (i49)*	Van Vliet Lake, WI	46.19211, -89.75436	Native	Crayfish Trap	07/6/2019	Male	53	✓	✓	✓	✓	OR93386971^am^; OR909905^bm^; OR909910^cm^
*Faxonius virilis* (i59)	South Turtle Lake, WI	46.21797, -89.89108	Native	Crayfish Trap	07/9/2019	Male	53	✓	✓	–	–	OR93386972^am^
*Pacifastacus gambelii* (i12)	Spirea Creek, WY	44.15546, -110.67996	Native	Kick Net	10/13/2020	Female	17	✓	–	–	–	OR93386983^am^
*Procambarus clarkii* (i62)	Chicago River, IL	41.97154, -87.70350	Invasive	Crayfish Trap	08/8/2019	Female	31	✓	–	–	–	OR93386982^am^
*Procambarus pictus* (i8)	North Fork Black Creek, FL	Undefined	Native	Kick Net	01/12/2021	Male	12	✓	✓	–	–	OR93386984^am^
*Procambarus pictus* (i13)	Lowry Lake, FL	Undefined	Native	Kick Net	03/9/2021	Female	12	✓	✓	–	–	OR93386985^am^
*Procambarus pictus* (i14)	Lowry Lake, FL	Undefined	Native	Kick Net	03/9/2021	Female	18	✓	✓	–	–	OR93386986^am^
*Procambarus pictus* (i22)	South Fork Black Creek, FL	Undefined	Native	Crayfish Trap	07/27/2021	Female	15	✓	✓	–	–	OR93386987^am^
*Procambarus pictus* (i23)	South Fork Black Creek, FL	Undefined	Native	Crayfish Trap	08/4/2021	Male	18	✓	✓	–	–	OR93386988^am^
*Procambarus pictus* (i3)	South Fork Black Creek, FL	Undefined	Native	Kick Net	09/27/2021	Female	20	✓	✓	✓	✓	OR93386989^am^; OR909906^bm^; OR909911^cm^
*Procambarus pictus* (i4)	South Fork Black Creek, FL	Undefined	Native	Crayfish Trap	02/11/2022	Female	22	✓	✓	–	–	OR93386990^am^
*Procambarus spiculifer* (i1)	South Fork Black Creek, FL	29.93819, -81.95489	Invasive	Crayfish Trap	09/11/2021	Male	47	✓	✓	✓	✓	OR93386991^am^; OR93386992^ag^; OR909907^bm^; OR909912^cm^
*Procambarus spiculifer* (i5)	South Fork Black Creek, FL	29.94124, -81.95655	Invasive	Crayfish Trap	09/11/2021	Male	29	✓	✓	–	–	OR93386993^am^
*Procambarus spiculifer* (i8)	South Fork Black Creek, FL	29.93819, -81.95489	Invasive	Crayfish Trap	09/11/2021	Female	22	✓	–	–	–	OR93386994^am^

a SSU accession number

b RPB1 accession number

c Hypothetical protein accession number

g Isolated from antennal gland

m Isolated from muscle

**Table 2:** Host samples used to attain the microsporidian genome data. Raw read data, overall resulting contigs, and assembly statistics (Quast v.5.0.2; Gurevich et al. 2013) are all provided.

Table 2					Assembly statistics			
Host	Sample type	Raw forward reads	Raw reverse reads	Total contigs (>500bp)	N50	N75	L50	L75
*Faxonius propinquus*	Muscle, DNA	2.4e^6^	2.6e^6^	189,576	775	602	61,615	118,694
*Faxonius virilis*	Muscle, DNA	2.5e^6^	2.8e^6^	300,728	841	630	97,904	186,243
*Faxonius rusticus*	Muscle, DNA	2.7e^6^	3.1e^6^	159,745	786	603	50,824	99,832
*Procambarus pictus*	Muscle, DNA	3.0e^6^	3.3e^6^	311,500	873	644	99,830	191,022
*Procambarus spiculifer*	Muscle, DNA	3.6e^6^	3.9e^6^	479,594	1029	725	146,546	282,677

**Table 3:** Morphological features of our novel *Nosema* isolates from each crayfish host compared to two described *Nosema* from crayfish hosts.

Taxa	*Nosema astafloridana* n. sp.				*Nosema rusticus* n. sp.		*Nosema wisconsinii* n. sp.				*Nosema austropotamobii* ^a^		*Nosema cheracis* ^b^	
Host	*Procambarus pictus*		*Procambarus spiculifer*		*Faxonius rusticius*		*Faxonius propinquus*		*Faxonius virilis*		*Austropotamobius pallipes*		*Cherax destructor*	
Spore shape	Pyriform		Pyriform		Pyriform		Pyriform		Pyriform		Pyriform		Pyriform	
Spore length (μm)	2.77 ±0.15	n=10	2.79 ±0.39	n=9	4.15 ±0.39	n=3	2.79 ±0.31	n=8	2.75 ±0.19	n=5	3.9 (3.5-4.3)^c^	n=50	3.4 (3.0-3.8)^c^	n=40
Spore width (pm)	1.49 ±0.13	n=10	1.58 ±0.25	n=9	1.90 ±0.19	n=4	1.57 ±0.05	n=8	1.49 ±0.13	n=5	2.2 (1.9- 2.5)^c^	n=50	1.9 (1.7-2.3)^c^	n=40
No. coils in polar filament	9-12		9-12		15-19		6-7		6-7		11-14		10-12	
Polar filament diameter (nm)	114 ± 12	n=10	121 ± 7	n=10	117± 13	n=10	109 ± 4	n=10	111 ± 5	n=10	74	n=20	82	n=50
Lateral exospore thickness (nm)	32 ± 5	n=10	24 ± 9	n=10	24 ± 4	n=10	27 ± 7	n=10	26 ± 4	n=10	34	n=10	31	n=10
Lateral endospore thickness (nm)	87 ± 11	n=10	87 ± 10	n=10	38 ± 7	n=10	88 ± 9	n=10	65 ± 14	n=10	54	n=10	52	n=10
Mature spore nucleus	Diplokaryotic		Diplokaryotic		Diplokaryctic		Unikaryotic		Unikaryotic		Unikaryotic		Unikaryotic	

a Pretto et al. 2018

b Moodie et al. 2003

c Light microscopy.

**Table 4:** General genomic details for the available *Nosema* genomes, including the newly sequenced genomes from crayfish-infecting *Nosema*.

Microsporidian	Host	Total contigs	Est. coverage	Cumulative haploid contig length (Mbp)	Number of annotated proteins	BUSCO score (%)	Reference
*Nosema wisconsinii* FP	*Faxonius propinquus*	542	20	5.42	2680	93.5	This study
*Nosema wisconsinii* FV	*Faxonius virilis*	534	19	5.41	2694	93.4	This study
*Nosema rusticus*	*Faxonius rusticus*	2545	80	7.77	3347	57.1	This study
*Nosema astafloridana* PP3	*Procambarus pictus*	767	35	7.05	3369	94.0	This study
*Nosema astafloridana* PS1	*Procambarus spiculifer*	628	12	7.05	3340	94.0	This study
*Nosema antheraeae*	*Antheraeae pernyi*	202	-	7.10	3863	91.8	SilkPathDB
*Nosema bombycis*	*Bombyx mori*	3558	-	14.40	4468	74.0	GCA000383075
*Nosema granulosis*	*Gammarus roeselii*	2007	65	8.80	3810	92.5	GCA015832245
