# Supplementary material for: Microsporidia and invertebrate hosts: genome-informed taxonomy surrounding a new lineage of crayfish-infecting Nosema spp. (Nosematida)
Source: Fungal Divers. Author manuscript; Available in PMC 2024 Nov 23. (PMC7616845; doi:10.1007/s13225-024-00543-w)
Supplement: Online Resources [file EMS200171-supplement-Online_Resources.zip › 13225_2024_543_MOESM1_ESM.docx]

**Microsporidia and invertebrate hosts: genome-informed taxonomy surrounding a new lineage of crayfish-infecting *Nosema* spp. (Nosematida)**

Cheyenne E. Stratton^1,*^, Sara A. Bolds^1,2^, Lindsey S. Reisinger^1^, Donald C. Behringer^1,3^, Amjad Khalaf^4^, Jamie Bojko^5,6,*^

^1^Fisheries and Aquatic Sciences, University of Florida, Gainesville, Florida, 32653, USA. ^2^School of Natural Resources, University of Florida, Gainesville, Florida, 32611, USA. ^3^Emerging Pathogens Institute, University of Florida, Gainesville, Florida, 32611, USA. ^4^Tree of Life, Wellcome Sanger Institute, Cambridge, CB10 1SA, UK. ^5^School of Health and Life Sciences, Teesside University, Middlesbrough, TS1 3BA, UK. ^6^National Horizons Centre, Teesside University, Darlington, DL1 1HG, UK.

Correspondence^*^: c.stratton@ufl.edu, J.Bojko@tees.ac.uk


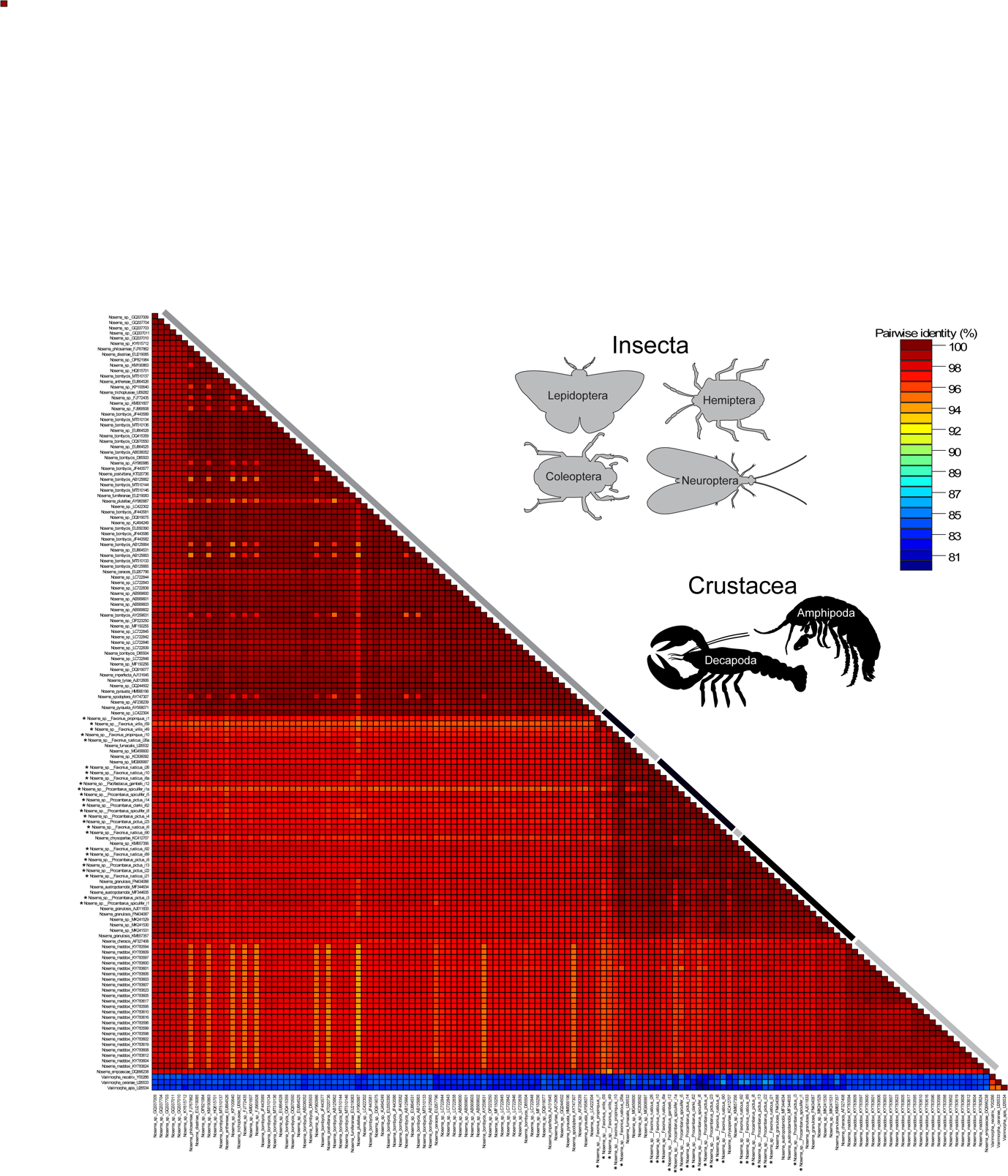


**Online Resource 1**. A similarity matrix illustrating the percent similarity of the rRNA gene of 140 *Nosema* isolates, including novel isolates presented in this study, and 3 *Vairimorpha* isolates. The host group each isolate belongs to is indicate by the gray (Insecta) or black (Crustacea) bars along the plot.


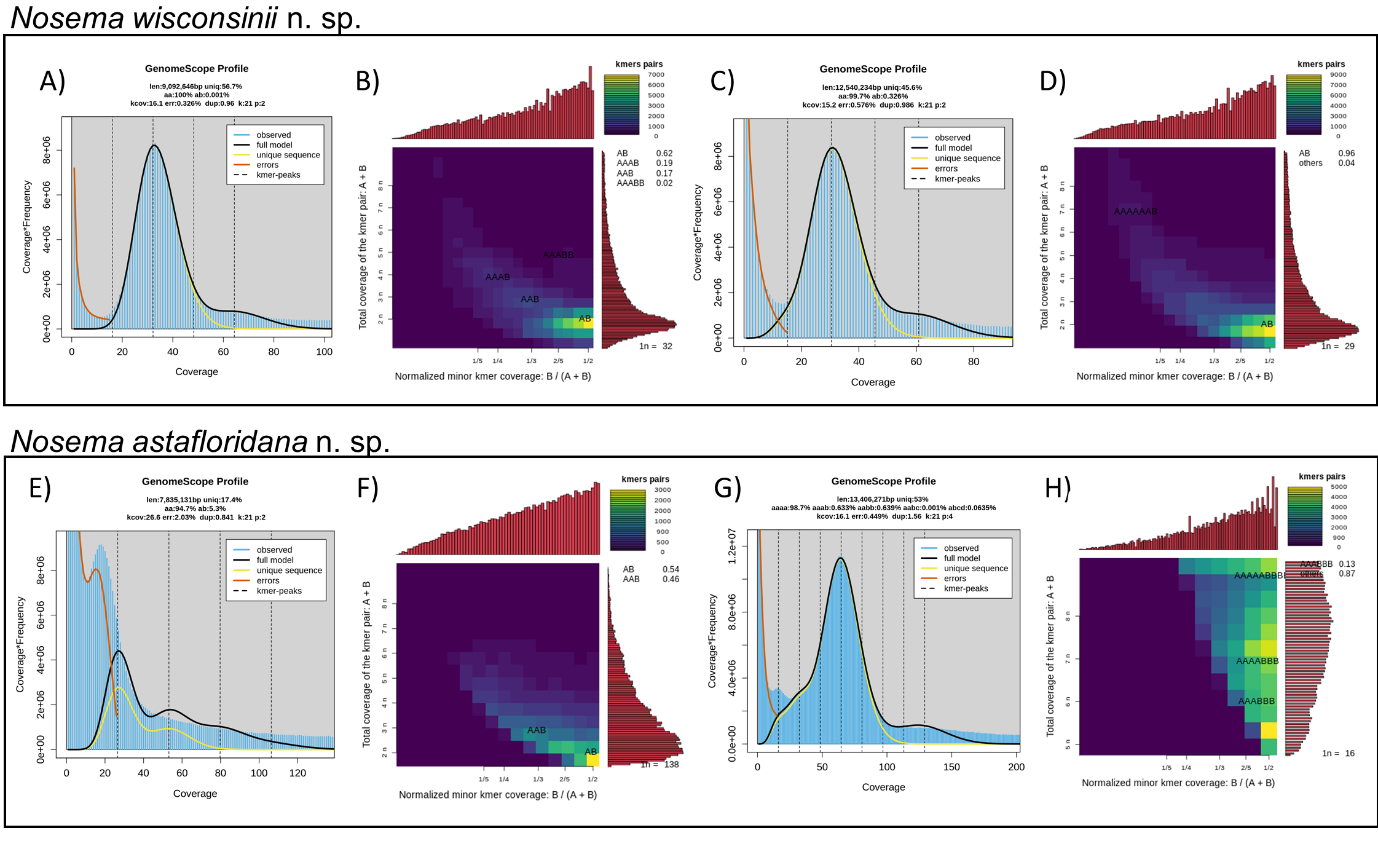


**Online Resource 3.** Ploidy estimate for *Nosema wisconsinii* n. sp. (A-D) and *Nosema astafloridana* n. sp. (E-H). (A) GenomeScope2 is the *N. wisconsinii* isolate from *Faxonius propinquus.* (B) Smudgeplot is the *N. wisconsinii* isolate from *F. propinquus*. (C) GenomeScope2 is the *N. wisconsinii* isolate from *Faxonius virilis*. (D) Smudgeplot is the *N. wisconsinii* isolate from *F. virilis*. (E) GenomeScope2 is the *N. astafloridana* isolate from *Procambarus spiculifer*. (F) Smudgeplot is the *N. astafloridana* isolate from *P. spiculifer*. (G) GenomeScope2 is the *N. astafloridana* isolate from *Procambarus pictus*. (H) Smudgeplot is the *N. astafloridana* isolate from *P. pictus*. The genome coverage for the isolates present was too low to determine ploidy confidently.

**
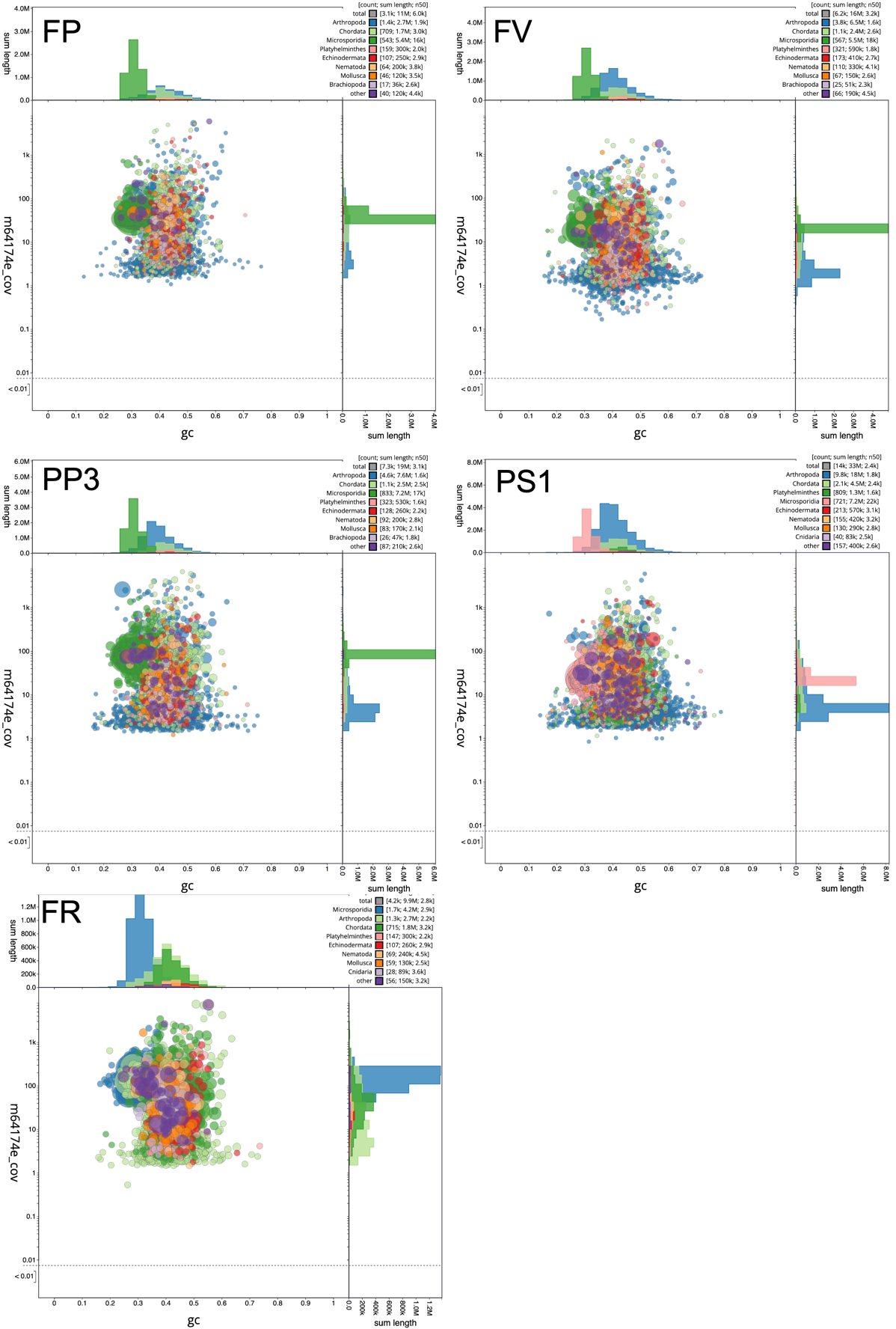
**

**Online Resource 4**. Blob plots from each crayfish host: *Faxonius propinquus* (FP), *Faxonius virilis* (FV), *Procambarus pictus* (PP3), *Procambarus spiculifer* (PS1), and *Faxonius rusticus* (FR). Each contig is mapped according to its coverage and GC content illustrating the starting assembly contains pathogen, host, and other aquatic organism DNA. A combination of BUSCO and Diamond/blastp was used to assign each contig to a taxonomic group which is coded by color on the plots.


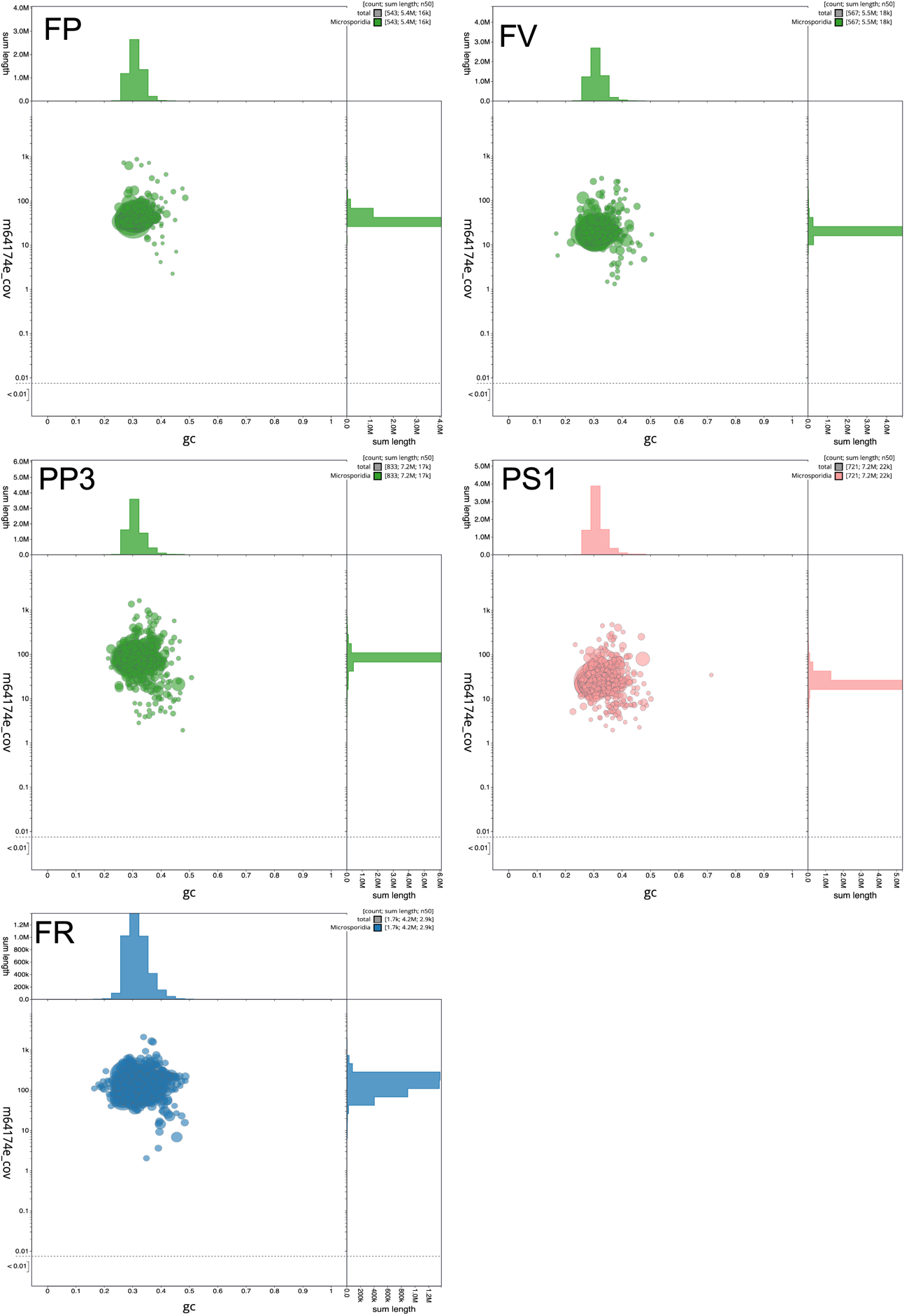


**Online Resource 5.** Blob plots from each crayfish host: *Faxonius propinquus* (FP), *Faxonius virilis* (FV), *Procambarus pictus* (PP3), *Procambarus spiculifer* (PS1), and *Faxonius rusticus* (FR). These plots only contain microsporidian-specific contigs. Each contig is mapped according to its coverage and GC content.
