# Supplementary material for: Microsporidia and invertebrate hosts: genome-informed taxonomy surrounding a new lineage of crayfish-infecting Nosema spp. (Nosematida)
Source: Fungal Divers. Author manuscript; Available in PMC 2024 Nov 23. (PMC7616845; doi:10.1007/s13225-024-00543-w)
Supplement: Online Resources [file EMS200171-supplement-Online_Resources.zip › 13225_2024_543_MOESM2_ESM.docx]

**Microsporidia and invertebrate hosts: genome-informed taxonomy surrounding a new lineage of crayfish-infecting *Nosema* spp. (Nosematida)**

Cheyenne E. Stratton^1,*^, Sara A. Bolds^1,2^, Lindsey S. Reisinger^1^, Donald C. Behringer^1,3^, Amjad Khalaf^4^, Jamie Bojko^5,6,*^

^1^Fisheries and Aquatic Sciences, University of Florida, Gainesville, Florida, 32653, USA. ^2^School of Natural Resources, University of Florida, Gainesville, Florida, 32611, USA. ^3^Emerging Pathogens Institute, University of Florida, Gainesville, Florida, 32611, USA. ^4^Tree of Life, Wellcome Sanger Institute, Cambridge, CB10 1SA, UK. ^5^School of Health and Life Sciences, Teesside University, Middlesbrough, TS1 3BA, UK. ^6^National Horizons Centre, Teesside University, Darlington, DL1 1HG, UK.

Correspondence^*^: c.stratton@ufl.edu, J.Bojko@tees.ac.uk

**1. Taxonomic Descriptions**

1.1 *Higher taxonomy*

Superphylum: Opisthosporidia (Karpov et al. 2014)

Phylum: Rozellomycota (Tedersoo et al. 2018), including Microsporidia (Balbiani, 1882; Wijayawardene et al. 2020)

Class: Terresporidia (Vossbrinck et al. 2014)

Order: Nosematida (Bojko et al. 2022; Wijayawardene et al. 2022)

1.2 *Microsporidian family*

Family: Nosematidae (Labbé, 1899; Tokarev et al. 2020)

“The family is defined as a monophyletic group including *Nosema* (type genus) and allied forms with small subunit ribosomal RNA gene sequence similarity over 77% both to *Nosema bombycis* (GenBank accession: D85503) and to *Vairimorpha necatrix* (GenBank accession: U11051). Developmental cycles are diverse. Conspicuous xenomas are usually not formed. Spores are ovoid or elongated oval. In most cases, the main sporulation sequence is disporoblastic and diplokaryotic (unikaryotic presporulation developmental stages may occur), and development is in direct contact with host cytoplasm. Binucleate or uninucleate mature spores with a thick spore wall and a polar tube with several coils in one to three ranks which are infectious per os to new hosts are produced. An early sporulation sequence may also occur in the life cycle, producing binucleate spores with a thinner spore wall and a shorter polar tube at the primary site of infection. Under certain environmental conditions, an additional octosporous sprogony takes place within the same host to produce eight sporoblasts within a sporophorous vesicle. Alternatively, the developmental stages are monokaryotic throughout the life cycle. Infections are typically systemic, although tissue tropism occurs in some species, and transmission can be transovarial, *per os* or both.” — Ammend. Tokarev et al. (2020)

1.3 *Microsporidian genus*

Genus: *Nosema* (Nageli, 1857; Tokarev et al. 2020)

“The genus description corresponds to that of the family. Small subunit ribosomal RNA gene sequence similarity to that of the type species, GenBank accession: D85503 is ≥ 94%. Ribosomal gene architecture is usually LSU-ITS-SSU. Type species: *Nosema bombycis* Nageli, 1857” — Tokarev et al. (2020)

1.4 *Nosema astafloridana* n. sp. Stratton, Bolds, Reisinger, Behringer, Khalaf, Bojko 2024

Species description: The microsporidian parasite infects the antennal gland and skeletal muscle of *P. spiculifer.* Merogony and sporogony take place within a sporophorous vesicle. Mature spores are pyriform in shape and measure 2.79 ± 0.39 (SD) μm in length and 1.58 ± 0.25 μm (SD) in width. The mature spore is diplokaryotic with 11-12 turns of the polar filament. Microsporidia within this species should have 98-100% similarity to the type specimen’s SSU and RPB1 loci (SSU accession: OR933891; RPB1 accession: OR909907; Genome accession: JBAOJC000000000; Bioproject number: PRJNA1076484).

Type host: *Procambarus spiculifer* (Cambaridae) Le Conte, 1856.

Type locality: South Fork Black Creek (29.93819, -81.95655) Bradford County, Florida, USA.

Site of infection: Antennal gland and skeletal muscle.

Etymology: The species ‘*astafloridana’* is named for the host taxa ‘*asta*’ (Astacoidea, referring to crayfishes) and the location in which it was found ‘*floridana*’ (Florida, USA).

Type material: Ethanol-fixed tissue, glutaraldehyde-fixed tissue, resin blocks, and histology slides are stored at the University of Florida, Reisinger Laboratory. Genetic data are deposited in NCBI under accession: OR933891 (SSU); OR909907 (RPB1); OR909912 (Hypothetical Protein). Genomic data are deposited JBAOJC000000000.

1.5 *Nosema rusticus* n. sp. Stratton, Bolds, Reisinger, Behringer, Khalaf, Bojko 2024

Species description: The microsporidian parasite infects the skeletal muscle and antennal gland of *F. rusticus*. Merogony and sporogony take place within a sporophorous vesicle. Mature spores are pyriform in shape and measure 4.15 ± 0.39 (SD) μm in length and 1.90 ± 0.19 (SD) μm in width. The mature spore is diplokaryotic with 15–19 turns of the polar filament. Microsporidia within this species should have 98-100% similarity to the type specimen’s SSU and RPB1 loci (SSU accession: OR933873; RPB1 accession: OR909903; Genome accession: JBAOJA000000000; Bioproject number: PRJNA1076482).

Type host: *Faxonius rusticus* (Cambaridae) Girard, 1852.

Type locality: Trout Lake (46.01913, -89.65534), Vilas County, Wisconsin, USA.

Site of infection: Antennal gland and skeletal muscle.

Etymology: The species is named for the host species (*Faxonius rusticus*) in which this novel species was found to infect.

Type material: Ethanol-fixed tissue, glutaraldehyde-fixed tissue, resin blocks, and histology slides are stored at the University of Florida, Reisinger Laboratory. Genetic data are deposited in NCBI under accession: OR933873 (SSU); OR909903 (RPB1); OR909909 (Hypothetical Protein). Genomic data are deposited JBAOJA000000000.

1.6 *Nosema wisconsonii* n. sp*.* Stratton, Bolds, Reisinger, Behringer, Khalaf, Bojko 2024

*Species description:* The microsporidian parasite infects the skeletal muscle of *F. virilis* and *F. propinquus*. Merogony and sporogony take place within a sporophorous vesicle. Mature spores are pyriform in shape and measure 2.75 ± 0.19 (SD) μm in length and 1.49 ± 0.13 (SD) μm in width. The mature spore is unikaryotic with 6–7 turns of the polar filament. Microsporidia within this species should have 98-100% similarity to the type specimen’s SSU and RPB1 loci (SSU accession: OR933871; RPB1 accession: OR909905; Genome accession: JBAOIZ000000000; Bioproject number: PRJNA1076481).

Type host: *Faxonius virilis* (Cambaridae) Hagen, 1870.

Type locality: Van Vliet Lake (46.192112, -89.754363), Vilas County, Wisconsin, USA.

Site of infection: Skeletal muscle.

Etymology: The species is named for the location (Wisconsin, USA) in which it was found.

Type material: Ethanol-fixed tissue, glutaraldehyde-fixed tissue, resin blocks, and histology slides are stored at the University of Florida, Reisinger Laboratory. Genetic data are deposited in NCBI under accession: OR933873 (SSU); OR909905 (RPB1); OR909910 (Hypothetical Protein). Genomic data are deposited JBAOIZ000000000.

**Supplementary References**

Balbiani E (1882) Sur Les Microsporidies Ou Psorospermies Des Articules. Compt Rend Acad Sco Paris. 95:1168-1171.

Bojko J, Reinke AW, Stentiford GD, Williams B, Rogers MS, Bass D (2022) Microsporidia: a new taxonomic, evolutionary, and ecological synthesis. Trends Parasitol 38:642-659.

Girard C (1852) A revision of the North American Astaci, with observations on their habits and geographical distribution. Proc Acad Nat Sci Phila 6:87-91.

Hagen HA (1870) Monograph of the North American Astacidae. Illustrated Catalogue of the Museum of Comparative Zoology at Harvard College 3:1-109.

Karpov SA, Mamkaeva MA, Aleoshin VV, Nassonova E, Lilje O, Gleason FH (2014) Morphology, phylogeny, and ecology of the aphelids (Aphelidea, Opisthokonta) and proposal for the new superphylum Opisthosporidia. Front Microbiol 5:112.

Labbé A (1899) Sporozoa. R. Friedländer und Sohn.

Le Conte J (1856) Descriptions of new species of *Astacus* from Georgia. Proc Acad Nat Sci Phila 7:400-402.

Nageli C (1857) *Nosema bombycis* Nageli. Botanische Zeitung 15:760-761.

Tedersoo L, Sánchez-Ramírez S, Kõljalg U, Bahram M, Döring M, Schigel D, May T, Ryberg M, Abarenkov K (2018) High-Level classification of the Fungi and a tool for evolutionary ecological analyses. Fungal Divers 90:135-159.

Tokarev YS, Huang WF, Solter LF, Malysh JM, Becnel JJ, Vossbrinck CR (2020) A formal redefinition of the genera *Nosema* and *Vairimorpha* (Microsporidia: Nosematidae) and reassignment of species based on molecular phylogenetics. J Invertebr Pathol 169:107279.

Vossbrinck CR, Debrunner-Vossbrinck BA, Weiss LM (2014) Phylogeny of the Microsporidia. In: Weiss LM, Becnel JJ (ed) Microsporidia: Pathogens of Opportunity. Wiley, pp 203-220.

Wijayawardene NN, Hyde KD, Al-Ani LKT et al (2020) Outline of Fungi and fungus-like taxa. Mycosphere 11:1060-1456.

Wijayawardene NN, Hyde KD, Dai DQ, Sánchez-García M, Goto BT, Magurno F (2022) Outline of Fungi and fungus-like taxa – 2021. Mycosphere 13:53-453.
