# Supplementary material for: Microsporidia and invertebrate hosts: genome-informed taxonomy surrounding a new lineage of crayfish-infecting Nosema spp. (Nosematida)
Source: Fungal Divers. Author manuscript; Available in PMC 2024 Nov 23. (PMC7616845; doi:10.1007/s13225-024-00543-w)
Supplement: Online Resources [file EMS200171-supplement-Online_Resources.zip › 13225_2024_543_MOESM5_ESM.docx]

**Microsporidia and invertebrate hosts: genome-informed taxonomy surrounding a new lineage of crayfish-infecting *Nosema* spp. (Nosematida)**

Cheyenne E. Stratton^1,*^, Sara A. Bolds^1,2^, Lindsey S. Reisinger^1^, Donald C. Behringer^1,3^, Amjad Khalaf^4^, Jamie Bojko^5,6,*^

^1^Fisheries and Aquatic Sciences, University of Florida, Gainesville, Florida, 32653, USA. ^2^School of Natural Resources, University of Florida, Gainesville, Florida, 32611, USA. ^3^Emerging Pathogens Institute, University of Florida, Gainesville, Florida, 32611, USA. ^4^Tree of Life, Wellcome Sanger Institute, Cambridge, CB10 1SA, UK. ^5^School of Health and Life Sciences, Teesside University, Middlesbrough, TS1 3BA, UK. ^6^National Horizons Centre, Teesside University, Darlington, DL1 1HG, UK.

Correspondence^*^: c.stratton@ufl.edu, J.Bojko@tees.ac.uk

Detailed comparative proteomics are described below. For each figure presented, the Pfam domains (confidence = >1.0e-20) present in the microsporidian species included on the x-axis are compared, according to Pfam groups associated with mitochondrial proteins. The comparison was made by downloading/annotating all available *Nosema* and *Vairimorpha* genomes and comparing the protein products using InterProScan v. 5.60-92.0. The ‘Freq’ key indicates the number of each domain that was detected from the annotated proteome. The specific Pfam in question is noted on the y-axis. The analysis includes proteins from the following genomes: *Nosema granulosis* (GCA_015832245), *Nosema bombycis* (GCA_000383075), *Nosema antheraeae* (SilkPathDB; PRJNA183977), *Vairimorpha ceranae* (GCF_000988165), *Vairimorpha* sp*. YNPr* (SilkPathDB; PRJNA325422), *Vairimorpha apis* (GCA_000447185) and *Vairimorpha* (=*Nosema*) *muscidifuracis* (GCA_028335825), in addition to those sequenced in this study.


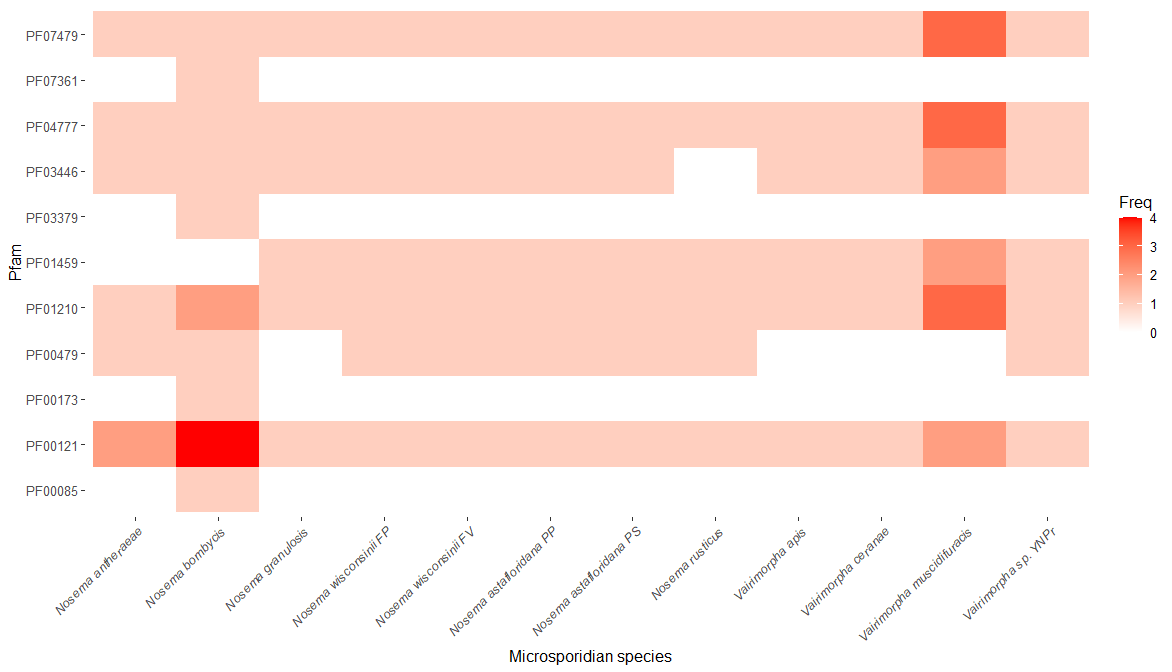


**Supplemental Figure 1.** Eleven mitochondria-associated Pfam domains were identified, in total, across the nine species. All of the microsporidians encoded proteins with PF07479, PF04777, PF01210, and PF00121 domains. PF00479 (Glucose-6-phosphate dehydrogenase) was encoded in all crayfish-infecting *Nosema*, and in the insect-infecting *Nosema*, but this domain was missing from all other isolates apart from the *Vairimorpha* sp. YNPr.


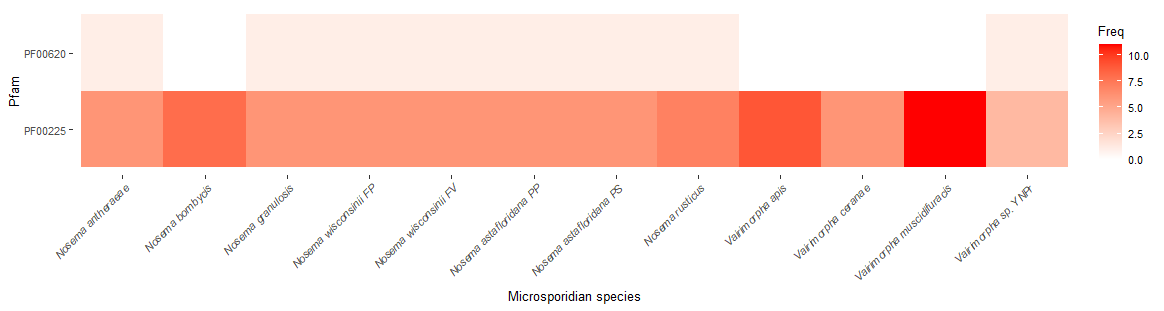


**Supplemental Figure 2.** Two motility-associated Pfam domains (PF00225, PF00620) were detected across the nine species. All were found to share PF00225 (kinesin), but the majority of *Nosema* encoded PF00620 (GTPase activated protein), in addition to *Vairimorpha* sp. YNPr.


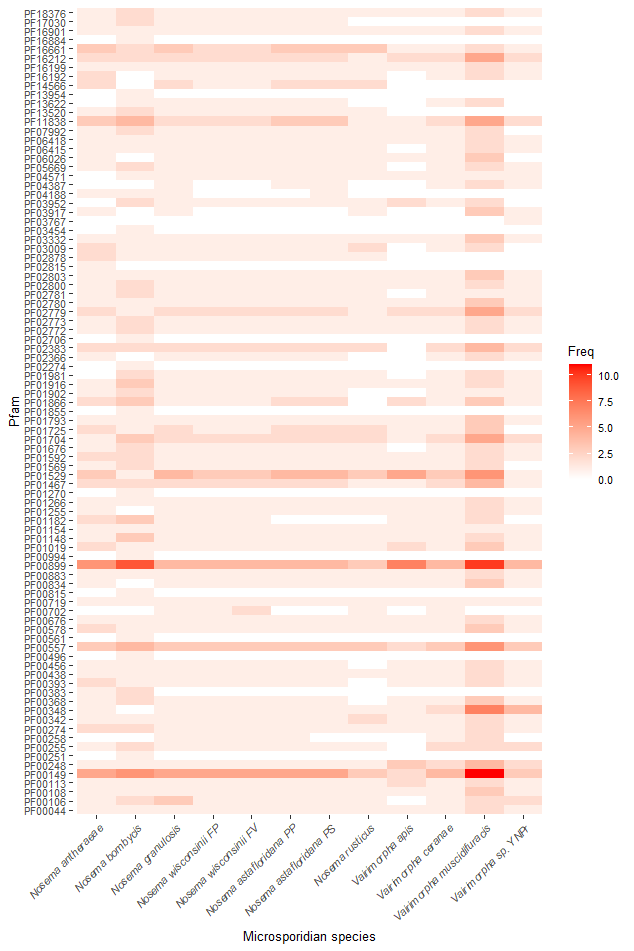


**Supplemental Figure 3.** Eighty-nine Pfam domains associated with general metabolism were identified in total. All of the *Nosema* spp. and *Vairimorpha* spp. shared 40 Pfam domains in total. PF17030 (microsporidia specific beta-lactamase) is missing in *N. rusticus* and all *Vairimorpha*, but present in all other *Nosema*. PF14566 (Inositol hexakisphosphatase - Phytase) is missing in *N. bombycis* but present in all other *Nosema*. PF02878 (alpha-D-phosphohexomutase) is present in all *Nosema* but missing from all *Vairimorpha.* Finally, PF13520 (Amino acid permease) appears to only be present in *Nosema*.


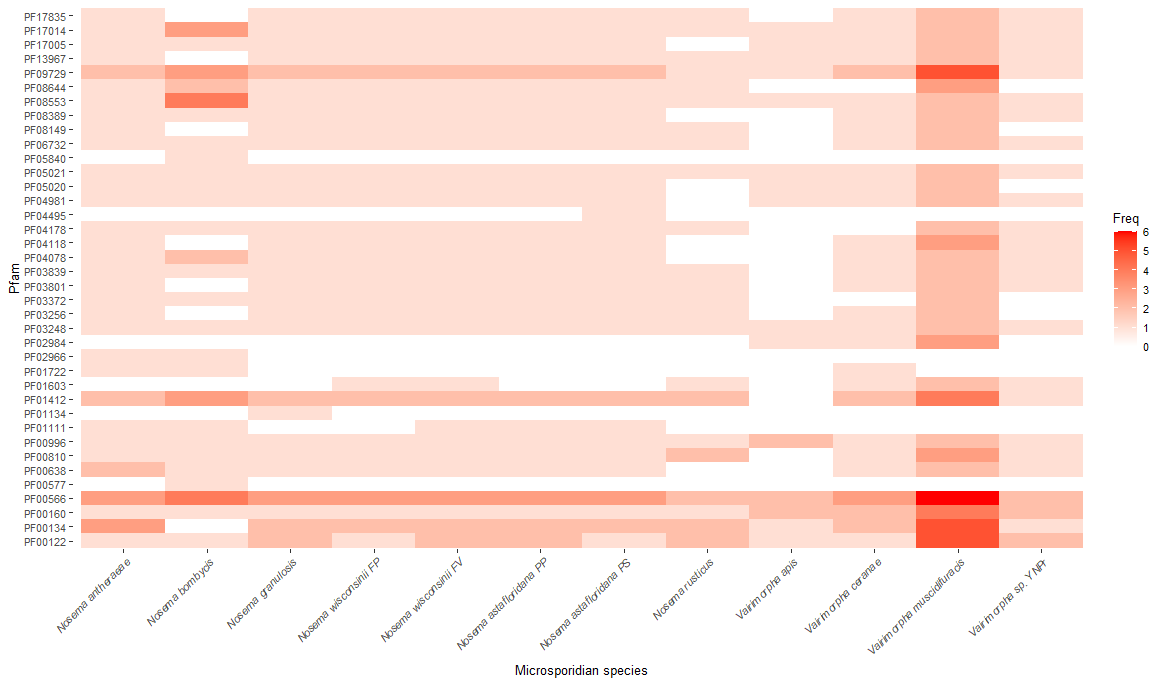


**Supplemental Figure 4.** Thirty-eight Pfam domains associated with the cell cycle were identified in total. Eight of these domains were shared across all of the taxa. PF01111 (cyclin-dependent) was only in *Nosema*, but it was missing from *N. granulosis* and our *N. wisconsinii* FP isolate. PF08644 (FACT complex) was found in all of the *Nosema*, in addition to *V. muscidifuracis*.


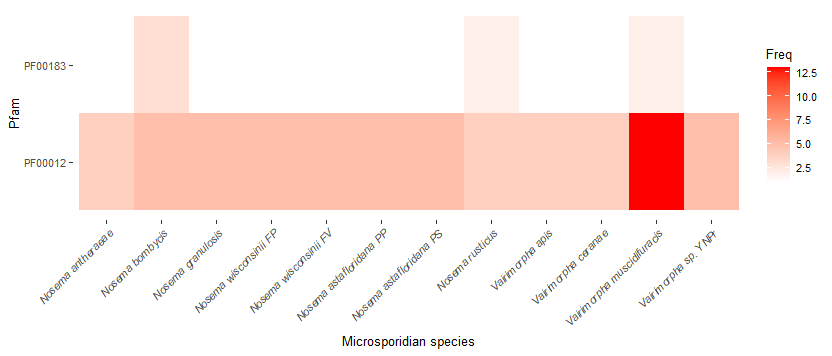


**Supplemental Figure 5.** Two Pfam domains encoded by shock proteins were identified across all taxa. All taxa encoded PF00012 (Hsp70); however, only *N. rusticus*, *N. bombycis* and *V. muscidifuracis* encoded PF00183 (Hsp90).


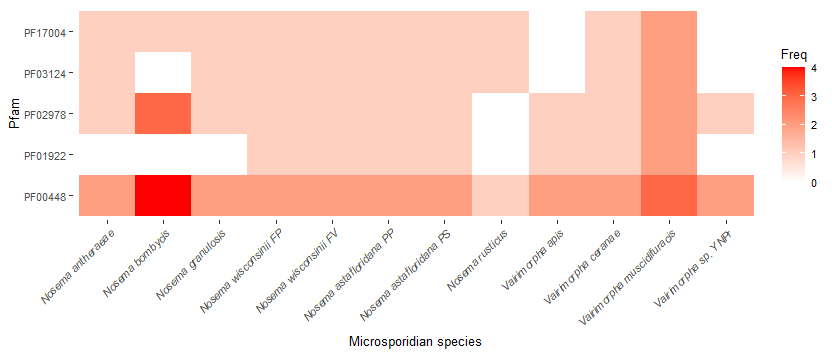


**Supplemental Figure 6.** Five Pfam domains associated with cell signaling were identified across all taxa. All taxa encoded PF00448 (P-loop NTPase), and only the crayfish-infecting *Nosema* encoded PF17004 (microsporidial recognition proteins) and PF03124 (signal transduction protein).


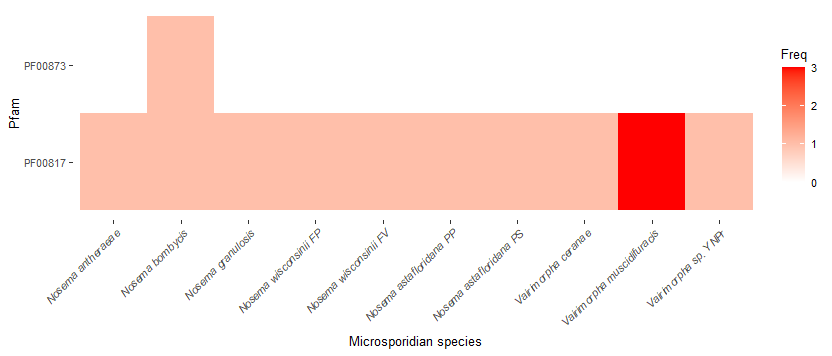


**Supplemental Figure 7.** Two Pfam domains associated with ultraviolet light (UV) protection were found. All of the microsporidians encoded PF00817 (impB/mucB/samB); however, only *N. bombycis* encoded PF00873. *Vairimorpha muscidifuracis* encoded 3 versions of PF00817 (impB/mucB/samB).


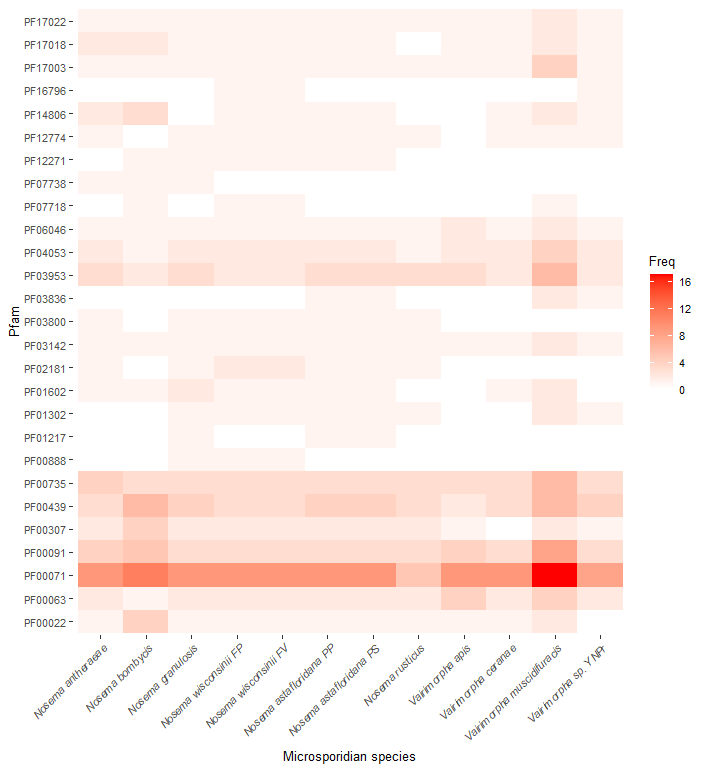


**Supplemental Figure 8.** Twenty-seven Pfam domains associated with structural function within the microsporidians were found across the taxa. Most *Nosema* encoded PF12271 (Chs7 membrane protein), PF03800 (mitotic spindle protein), and PF02181 (Formin), which appear to be missing from the *Vairimorpha*. *Nosema astafloridana* and *Nosema granulosis* both encode PF01217 (sigma/mu subunit), where the others do not.

**
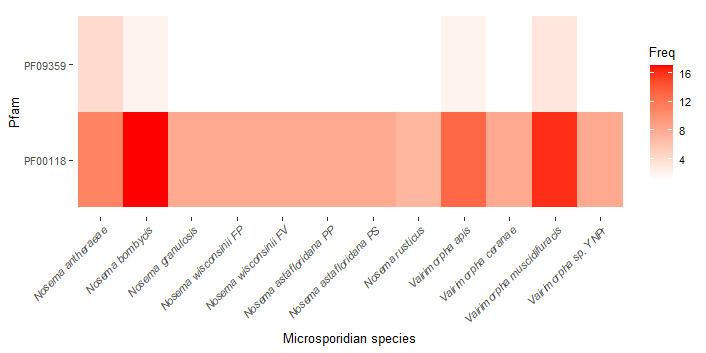
**

**Supplemental Figure 9.** Two additional chaperone proteins unassigned to other groups were identified across the taxa. All of the taxa included shared PF00118 (TCP-1); however, the aquatic microsporidians were found to be missing PF09359 (vacuolar transport chaperone).


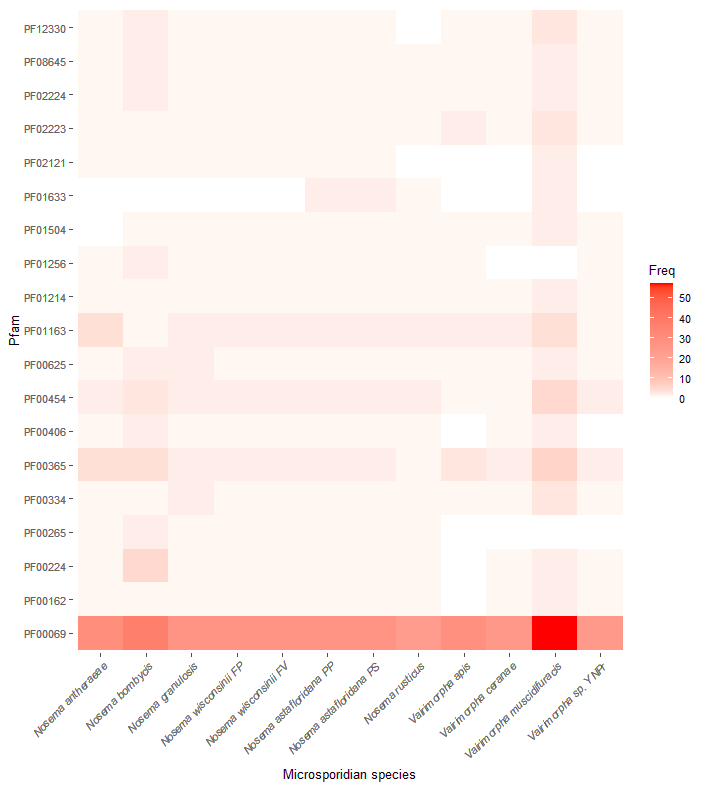


**Supplemental Figure 10.** Nineteen Pfam domains associating with kinase activity were documented across the taxa. Of these, only the *Nosema* encoded PF00265 (Thymidine kinase), which was missing from all of the *Vairimorpha*.

**
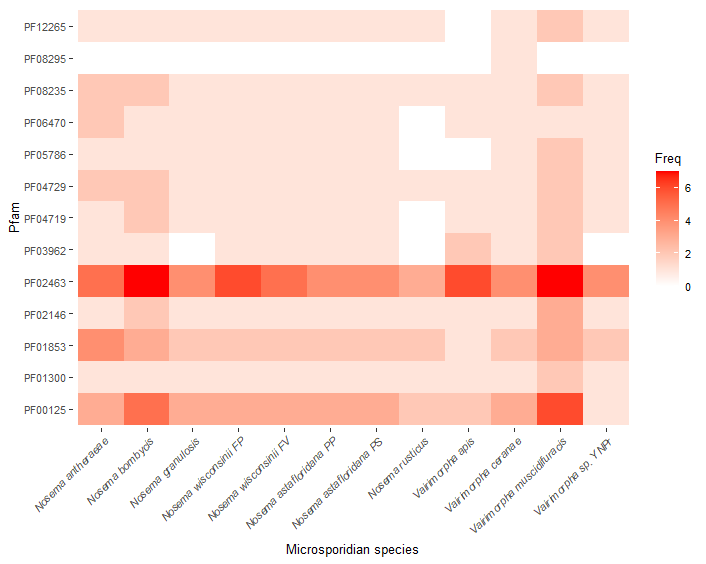
**

**Supplemental Figure 11.** Thirteen Pfam domains associated with chromosomal maintenance were found across taxa. Generally, all of the Pfam domains were shared by all taxa; however, *V. muscidifuracis* encoded a unique Pfam – PF08295 (associated with histone deacetylases).


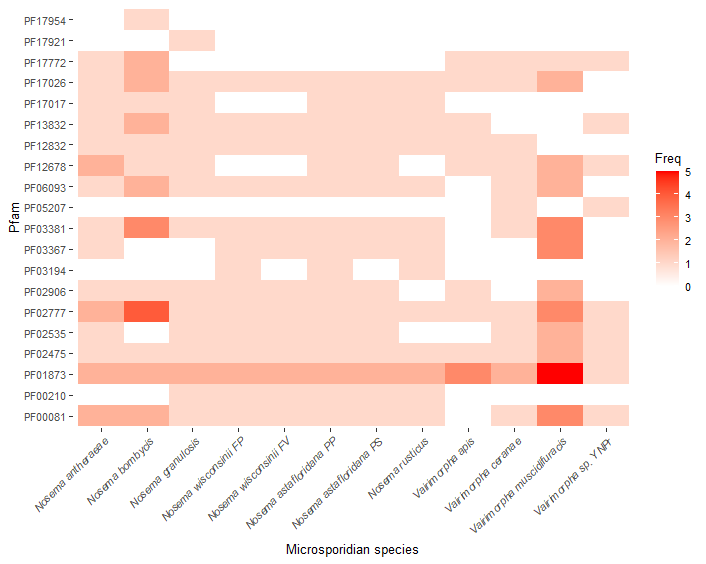


**Supplemental Figure 12.** Twenty Pfam domains that are associated with metal ion binding properties were found across the taxa. The metal binding Pfam PF05207 (zinc binding motif) was restricted to the *Vairimorpha*. PF00210 (Ferritin) was only present in the aquatic *Nosema*. PF17017 (microsporidia-specific zinc-finger motif) was found only in the *Nosema*, but it was missing from one crayfish-infecting species. PF03194 (snRNA/splicing associated) was present only in the crayfish-infecting *Nosema*.


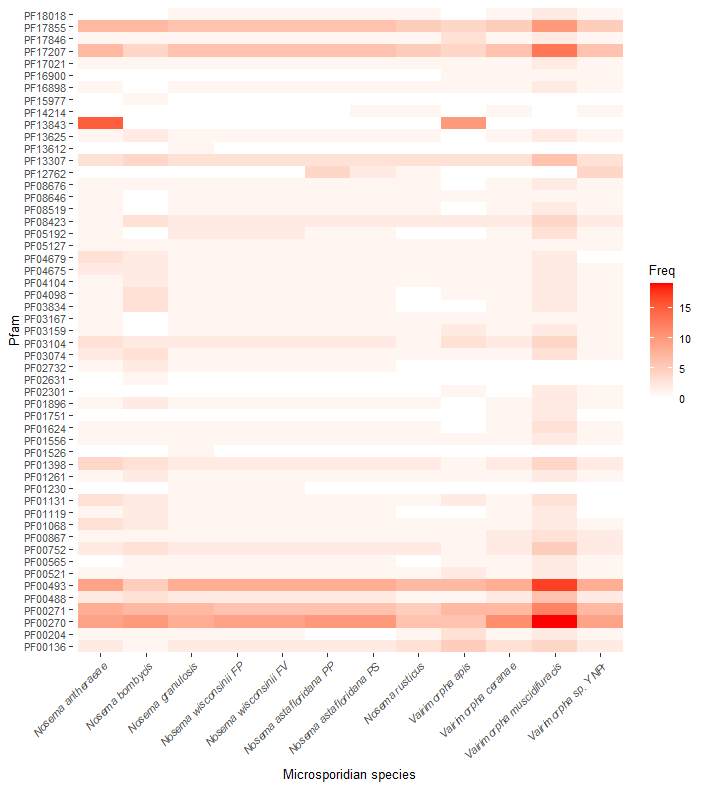


**Supplemental Figure 13.** Fifty-three Pfam domains associated with DNA replication and repair were noted. Largely, these Pfam domains were shared by all taxa; however, PF02732 (nuclease) was shared only among the *Nosema.*

**
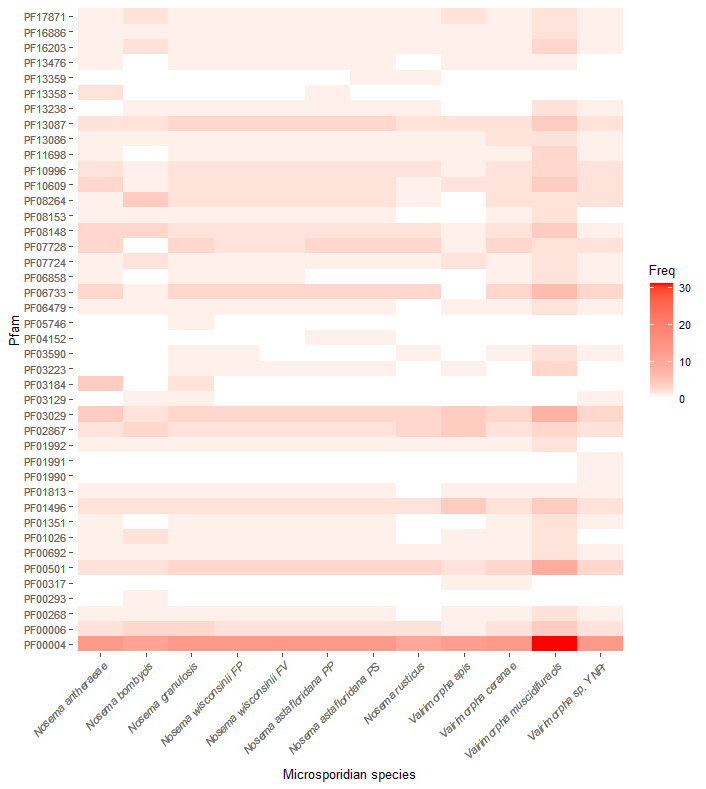
**

**Supplemental Figure 14.** Forty-two Pfam domains associated with nucleotide synthesis and catalysis were largely shared across the taxa. However, *N. astafloridana* uniquely encoded PF04152 (multi-subunit nuclease) and *N. rusticus* and *N. astafloridana* (PS isolate) encoded PF13359 (DDE superfamily of nucleases).

**
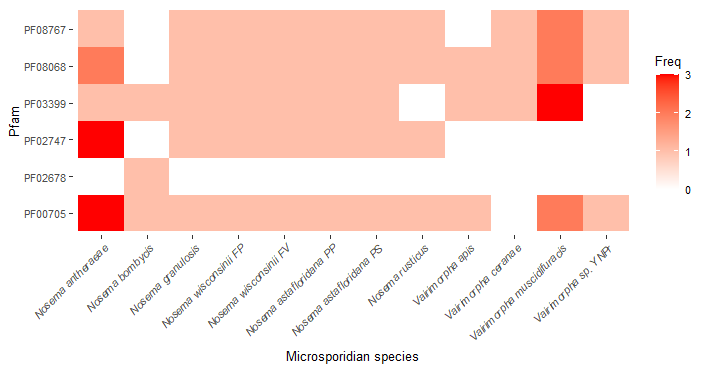
**

**Supplemental Figure 15.** Six Pfam doamins were related to nuclear localization.


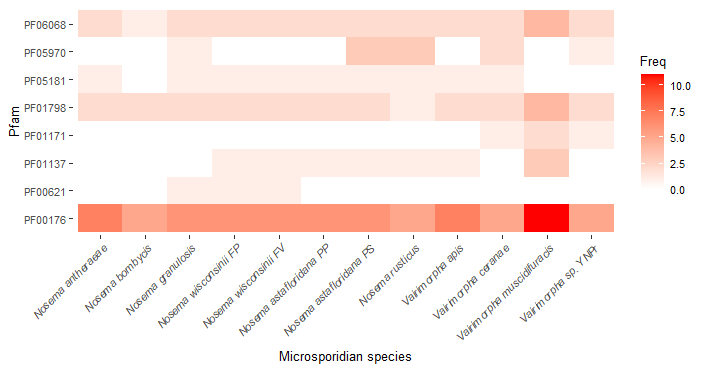


**Supplemental Figure 16.** Eight Pfam doamins were associated with nucleotide binding. *Nosema* *granulosis* and *N. wisconsinii* both encode PF00621 (Guanine nucleotide exchange factor), which is missing in the other taxa. PF01171 (PP-loop superfamily) is only encoded by the *Vairimorpha*. Three domains (PF06068, PF01798, PF00176) are shared among all the taxa.


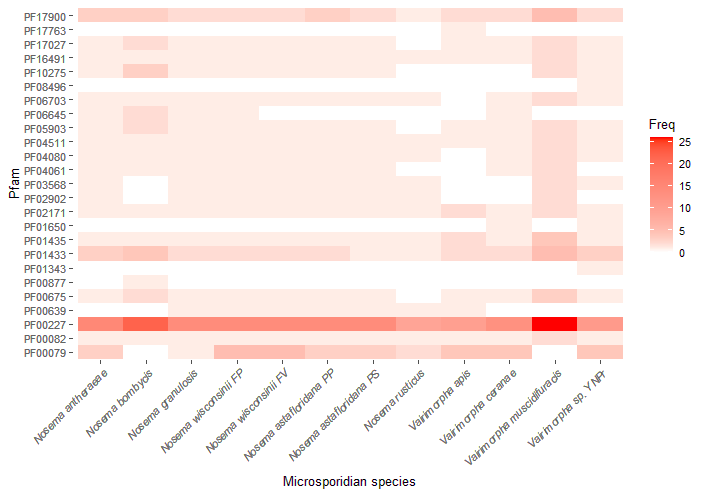


**Supplemental Figure 17.** Twenty-five Pfam domains were associated with protein synthesis, folding, and/or catalysis. Only the *Vairimorpha* encoded PF01650 (Asparaginyl endopeptidase), and the majority were shared among all the isolates.


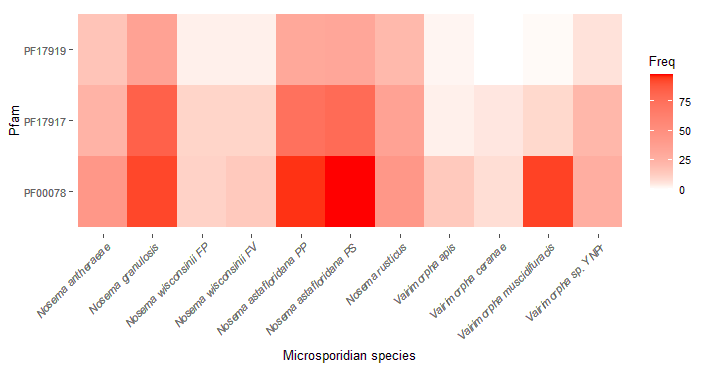


**Supplemental Figure 18.** Three Pfam domains were linked with reverse transcription activity. The number of times these Pfams were detected varied among the different taxa (Fig. 10). There is just one instance of a species missing a Pfam, which involves *V. ceranae*, which is missing PF17919 (a ribonuclease associated with reverse transcription).


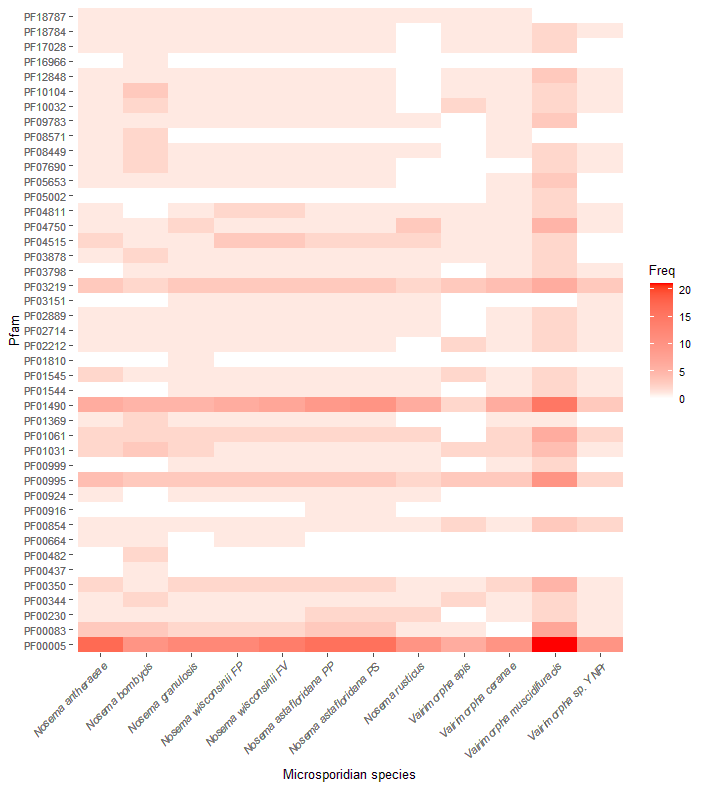


**Supplemental Figure 19.** Forty-three Pfam domains related to secretion systems in the microsporidians checked. PF00924 (mechanosensitive channel) was only present in the *Nosema*, where PF05002 (calcyclin binding) was only found in the *Vairimorpha*. PF00664 (transmembrane helices) was only noted in *N. wisconsinii* and the terrestrial *Nosema* spp., where PF00916 (Sulfate Permease) was found only in *N. astafloridana*.

**
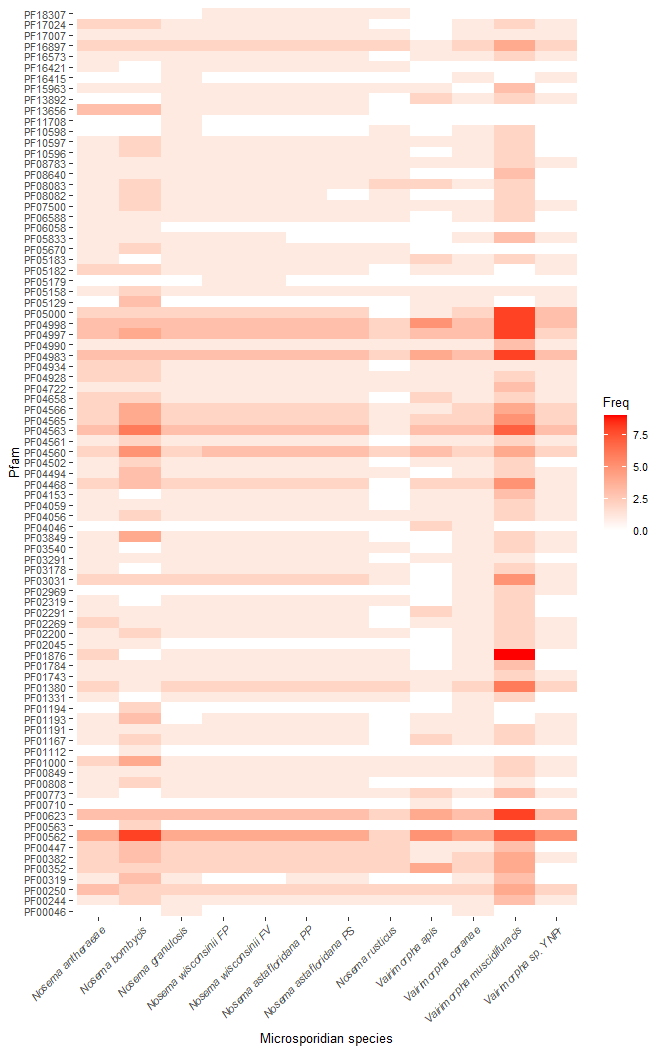
**

**Supplemental Figure 20.** Eighty-five Pfam domains related broadly to transcription were found across the taxa. PF18307 (transcription factor) was only identified from crayfish-infecting *Nosema*, in addition to PF05179 (RNA polymerase II accessory factor), which was only noted from *N. wisconsinii*. PF16421 (coiled coil domain of E2F transcription factors) was present in all of the *Nosema*, except *N. bombycis*, and not in the *Vairimorpha*. PF13656 (Rpb3 and Rpb11 assembly domain) was also found in all of the *Nosema*, but not *N.* *rusticus* or *Vairimorpha*. PF00808 (histone-like transcription factor) was found in all *Nosema*, and in low relative number in *V. muscidifuracis*. Finally, PF06058 (Dcp1-like de-capping enzyme) was only documented from the terrestrial, insect-infecting, *Nosema*.


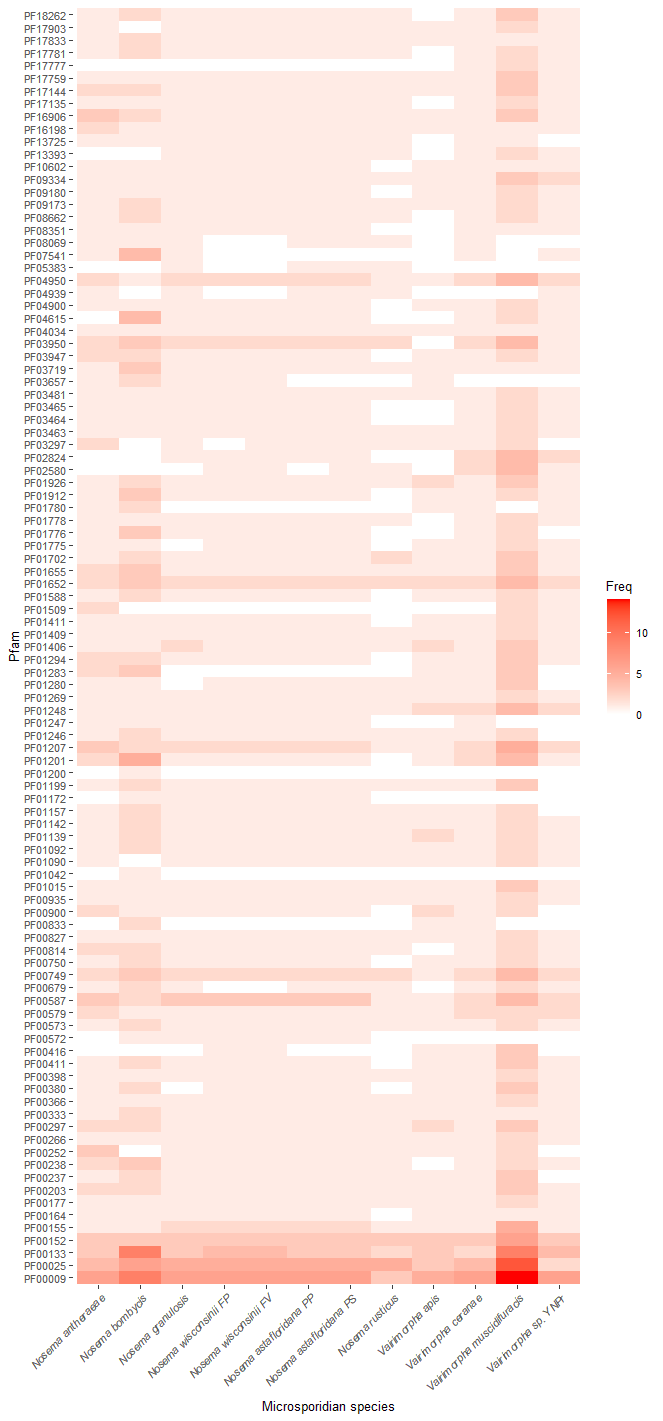


**Supplemental Figure 21.** One-hundred-and-one Pfam domains associated with the general process of translation were identified. PF17777 (ribosomal L10-associated) was only present in the *Vairimorpha* spp. and both PF01172 (Shwachman-Bodian-Diamond syndrome-related) and PF00572 (Ribosomal protein uL13-related) were present in most *Nosema* and missing from the *Vairimorpha*. PF01780 (Ribosomal L37ae-related) and PF01283 (ribosomal subunit protein eS26-like) were missing from all aquatic *Nosema*.

**
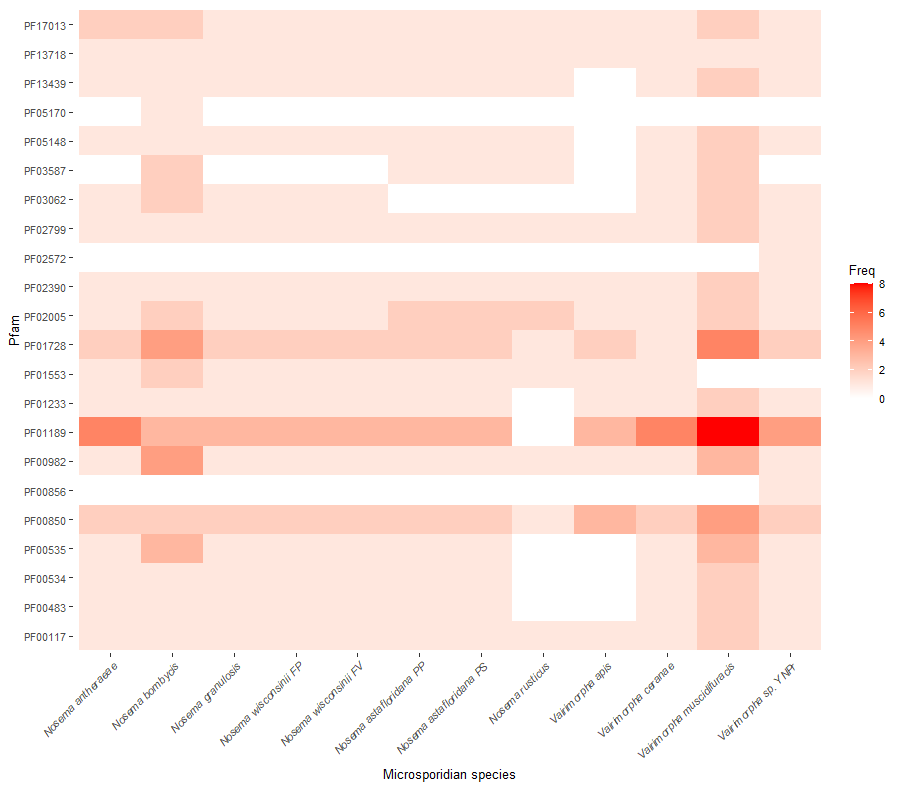
**

**Supplemental Figure 22.** Twenty-two Pfam domains were generally associated with transferase activity. Eight were shared by all of the taxa, otherwise their presence did not show a discernable pattern.

**
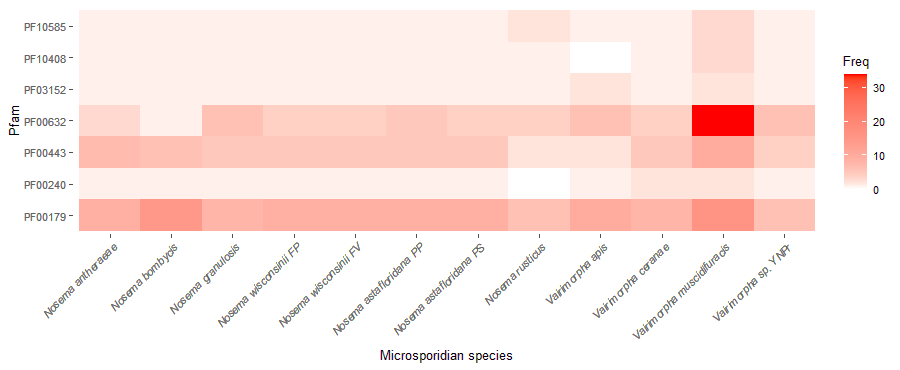
**

**Supplemental Figure 23.** Seven Pfam domains associated with ubiquitination were shared by all of the taxa, otherwise their presence did not show a discernable pattern.


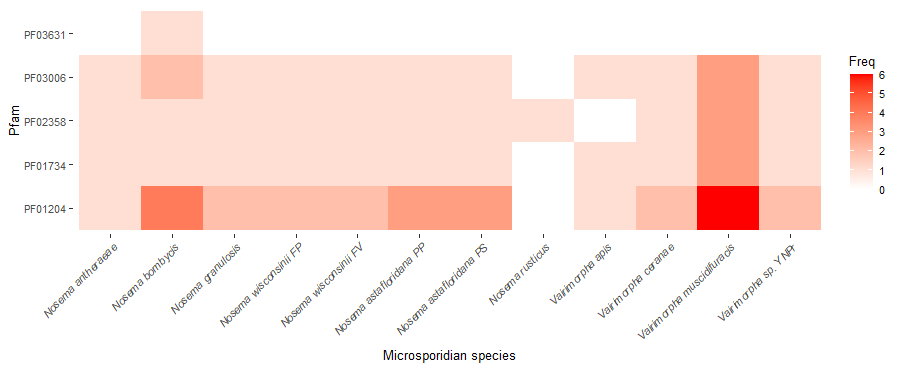


**Supplemental Figure 24.** Five Pfam domains that are thought to be related to virulence were present across the taxa, apart from *N. rusticus*, which exhibited only one of these Pfam domains (PF02358).
