# Supplementary material for: Microsporidia and invertebrate hosts: genome-informed taxonomy surrounding a new lineage of crayfish-infecting Nosema spp. (Nosematida)
Source: Fungal Divers. Author manuscript; Available in PMC 2024 Nov 23. (PMC7616845; doi:10.1007/s13225-024-00543-w)
Supplement: Online Resources 2, 6, 7 and 8 Legend [file EMS200171-supplement-Online_Resources_2_6_7_and_8_Legend.docx]

**Supplementary Material**

Online Resource 2. Taxonomic description of our three novel microsporidian species: *Nosema astafloridana* n. sp.; *Nosema rusticus* n. sp.; *Nosema wisconsinii* n. sp.

Online Resource 6. A list of all single copy Pfams found by InterProScan v. 5.60-92.0 with information on what the Pfam codes for.

Online Resource 7. The data used to create the heat maps (Fig. 10; Online Resources8-31) to compare the number of available predicted domains of each Pfam within and across the *Nosema* and *Vairimorpha*. The comparison was made by downloading/annotating all available *Nosema* and*Vairimorpha* genomes and comparing the protein products using InterProScan v. 5.60-92.0. The analysis includes proteins from the following genomes:*Nosema granulosis* (GCA_015832245)*, Nosema bombycis*(GCA_000383075)*, Nosema antheraeae*(SilkPathDB; PRJNA183977)*, Vairimorpha ceranae* (GCF_000988165)*, Vairimorpha*sp*.* YNPr(SilkPathDB; PRJNA325422), *Vairimorphamuscidifuracis* (GCA_028335825), and *Vairimorphaapis* (GCA_000447185), in addition to those sequenced in this study.

Online Resources8-31. Supplemental Figures and Captions for each individualPfam group.
